# The neurophysiological correlates of altruism: A scoping review of fMRI, EEG, and autonomic studies

**DOI:** 10.3758/s13415-025-01380-3

**Published:** 2025-12-17

**Authors:** Junya Orui, Takao Inoue, Keigo Shiraiwa, Masaya Ueda, Yasuo Naito, Ryouhei Ishii

**Affiliations:** 1 Department of Health Science, Osaka Health Science University, 1-9-27 Tenma, Osaka, Kita-ku 530-0043 Japan; 2 Department of Occupational Therapy, Osaka Metropolitan University Graduate School of Rehabilitation Science, 2-1-132, Morinomiya, Osaka, Joto-ku 536-8525 Japan; 3 Department of Rehabilitation, Osaka Kawasaki Rehabilitation University, 158 Mizuma, Osaka, Kaizuka 597-0104 Japan; 4 Department of Psychiatry, The University of Osaka Graduate School of Medicine, 2-2 Yamadaoka, Osaka, Suita 565-0871 Japan

**Keywords:** Altruism, FMRI, EEG, Autonomic, Social cognition

## Abstract

Altruistic motivation, a fundamental aspect of human behavior, commands significant neuroscience attention. This scoping review, following PRISMA-ScR guidelines, synthesizes findings from 45 fMRI, EEG, fNIRS, and autonomic nervous system (ANS) studies published between 1975 and 2024, exploring the neural basis of costly, other-oriented altruism. Our review covers experimental paradigms utilizing fMRI, EEG, and autonomic measures to interrogate neural and physiological responses associated with altruistic decision making and behavior. Importantly, inclusion was not limited to online, state-dependent, or event-related measurements; studies employing various acquisition timings were encompassed, as long as they examined neural or physiological correlates of altruistic motivation. fMRI studies consistently reveal engagement of a complex neural network, including medial and dorsolateral prefrontal cortex, temporoparietal junction, insula, and anterior cingulate cortex, integrating social cognition, value-based decision making, and emotional processing inherent in altruistic acts. EEG research highlights increased left frontal activity, indicative of approach-oriented motivation, and frontal midline theta, reflecting cognitive control during altruistic choices. ANS findings exhibit variability but tentatively suggest parasympathetic dominance in altruistic context. A key contribution is a multi-stage information-processing model of altruistic motivation, integrating these modalities from socio-affective appraisal through value computation, motivational orientation, and cognitive control to behavioral implementation. Findings elucidate the interplay of cognitive and emotional processes driving altruism, offering a nuanced conceptual framework. Future research, including longitudinal studies, cross-cultural comparisons, and ecologically valid assessments, is vital for advancing theoretical and practical understanding of the neurophysiology of altruism.

**Supplementary Information:**

The online version contains supplementary material available at 10.3758/s13415-025-01380-3.

## Introduction

Altruism, a fundamental aspect of human sociality, is broadly characterized by actions undertaken to benefit others, often at some cost to oneself (Batson, 2011; Fehr & Fischbacher, 2003). It manifests in a diverse spectrum of behaviors, ranging from significant commitments like charitable donations and volunteering to quotidian acts of kindness and instrumental helping (Lay & Hoppmann, 2015). Elucidating the origins, motivations, and underlying mechanisms of altruism has long been a central pursuit across disparate academic disciplines, including evolutionary biology, psychology, economics, and, more recently, cognitive and social neuroscience (Hu et al., 2023). Altruism has profound societal implications, ranging from fostering robust interpersonal relationships and individual well-being to ensuring societal cohesion (Post, 2005; Rahimyar & Sarvari, 2023; Stocks & Lishner, 2023). Consequently, gaining a deep understanding of its neurobiological underpinnings is not merely an academic curiosity but a critical endeavor. Crucially, the conceptualization and operationalization of altruism vary considerably across these fields, making definitional clarity essential when investigating its neurobiological underpinnings (Clavien & Chapuisat, 2013).

From an evolutionary biology standpoint, altruism is typically defined by its consequences for reproductive fitness: an organism’s behavior is considered altruistic if it diminishes its own fitness while concurrently enhancing the fitness of another (Fetchenhauer & Bierhoff, 2004; West et al., 2007). In stark contrast, psychological perspectives primarily emphasize the motivational state propelling the behavior. Psychological altruism is thus defined as a “motivational state with the ultimate goal of increasing another’s welfare” (Batson, 2011; Filkowski et al., 2016). This is meticulously distinguished from psychological egoism, where the ultimate aim is to augment one’s own welfare, even if benefiting others serves as an instrumental means to that end (Narveson, 2012). The empathy-altruism hypothesis, a prominent psychological model, posits that empathic concern elicited by witnessing another’s distress evokes a genuinely altruistic motivation to alleviate their suffering (Batson et al., 1981; Schroeder et al., 2015). Behavioral economics frequently examines altruistic behavior through choices in structured experimental games (e.g., Dictator Game, Prisoner’s Dilemma, Public Goods Game), which assess constructs such as fairness, cooperation, and costly helping (Camerer, 2010; Lazić et al., 2021). These studies often fall under the broader rubric of “prosocial behavior,” an umbrella term encompassing any action intended to benefit other individuals or society at large (e.g., helping, sharing, cooperating, comforting) (Bellucci et al., 2020; Eisenberg & Miller, 1987). While prosocial behavior can indeed be driven by genuinely altruistic motives, it can also emanate from a variety of other motivations, including reciprocity, conformity, reputation management, or the pursuit of social approval (Pfattheicher et al., 2022).

The advent and rapid advancement of social and cognitive neuroscience have opened unprecedented avenues for exploring the brain mechanisms subserving altruistic and prosocial acts (Bellucci et al., 2020; San Martín et al., 2016; Wu & Hong, 2022). Methodologies such as functional magnetic resonance imaging (fMRI), electroencephalography (EEG), functional near-infrared spectroscopy (fNIRS), and autonomic nervous system (ANS) measures are increasingly employed to delineate the neural and physiological correlates of decisions to help, donate, or cooperate, often when such actions entail personal cost. However, a coherent synthesis of findings across this burgeoning and methodologically diverse literature requires a precise and consistently applied operational definition of the phenomenon under investigation. While evolutionary concepts furnish an ultimate causal framework, neuroscientific inquiries typically concentrate on the proximate psychological and neural mechanisms engaged when individuals contemplate and execute decisions intended to benefit others. For this scoping review, which aims to systematically map and synthesize findings from fMRI, EEG, fNIRS, and ANS studies in humans, “altruistic motivation” is defined as the underlying psychological impetus to increase another’s welfare, and the resulting behavior often involves some tangible cost (e.g., monetary, time, physical effort, social risk) to the actor. This definition aligns closely with psychological altruism and was specifically chosen to effectively capture the core of altruistically motivated prosocial behaviors as they are commonly operationalized in the neuroimaging and psychophysiological literature that underpins this review. We employ this definition to clearly distinguish our focus from (a) broader conceptualizations of prosocial behavior that may lack a direct other-oriented motivational core (e.g., rule compliance or cooperation driven purely by strategic self-interest) and (b) purely evolutionary definitions centered on ultimate fitness consequences rather than proximate mechanisms. Establishing this conceptual clarity is paramount not only for achieving a coherent synthesis but also for isolating the specific neurobiological signatures of intrinsically other-oriented drive, a distinction often blurred in studies examining prosociality more broadly.

Previous meta-analyses and reviews have examined the neural correlates of related, but distinct, concepts. For instance, Boccadoro et al. (2021) focused on altruistic punishment, while Cutler and Campbell-Meiklejohn (2019) conducted an influential meta-analysis of fMRI studies on “giving,” which acknowledged the inclusion of both genuinely altruistic and more strategically motivated donations. A comprehensive meta-analysis by Bellucci et al. (2020) investigated prosociality more broadly, linking it to established mentalizing and empathy networks but also highlighting distinct neural activations associated with different facets of prosocial behavior. However, despite these valuable contributions, a focused scoping review that specifically integrates findings related to altruistic motivation (as defined above) across the complementary modalities of fMRI, EEG, fNIRS, and ANS measures was still needed. Such an endeavor is essential because focusing on this precisely defined motivational state, rather than broader prosociality or heterogeneous “giving” behaviors, allows for a more nuanced understanding of the core neural mechanisms that drive intrinsically other-regarding actions. Furthermore, while these prior works are foundational, they do not offer a specific synthesis that simultaneously considers evidence from these four distinct neurophysiological modalities, each providing unique and synergistic insights: fMRI offering high spatial resolution for network localization, EEG providing superior temporal resolution for dynamic processing, fNIRS balancing ecological validity with cortical activity measurement, and ANS measures capturing crucial peripheral physiological responses indicative of affective and motivational states. While compassion, often considered a potent precursor to altruistic action, has been linked to specific autonomic changes such as decreased heart rate (Eisenberg et al., 1989) or heightened parasympathetic activity (Correa et al., 2015; Goetz et al., 2010; Stellar et al., 2015), a systematic integration of these peripheral physiological findings within the broader neurobiological framework of our specific construct of altruistic motivation remains conspicuously lacking. This omission is significant, as a holistic understanding of altruism necessitates bridging central neural processes with their peripheral physiological manifestations. Gaining a more integrated understanding of the multi-level neurophysiological mechanisms underlying altruism is critically important, not only for advancing basic science but also because altruistic behavior is inextricably linked to the quality of interpersonal relationships, individual mental health and well-being, and the overall cohesion and functionality of society (Post, 2005; Rahimyar & Sarvari, 2023; Stocks & Lishner, 2023). Furthermore, a growing body of evidence suggests that altruistic tendencies and related socio-emotional capacities can be cultivated and enhanced through targeted training programs (Böckler et al., 2018; Klimecki et al., 2014; Weng et al., 2013), underscoring the potential societal relevance and translational implications of this line of inquiry.

This scoping review aims to address the identified gap in the literature by systematically searching, identifying, mapping, and synthesizing empirical studies that have measured brain activity (using fMRI, EEG, or fNIRS) and/or ANS activity in relation to altruistic motivation in human participants. Our primary objectives are to: (1) provide a comprehensive and nuanced overview of the neurophysiological correlates consistently (and inconsistently) associated with our specific definition of altruistic motivation across diverse measurement modalities and experimental paradigms; (2) identify key consistencies, discrepancies, and methodological variations in the existing findings, thereby highlighting robust patterns as well as areas requiring further investigation to advance the field beyond current, sometimes fragmented, understanding; and (3) propose a synthesized, integrated heuristic model of the neural and physiological processes that may underpin altruistic motivation and decision making. This heuristic framework, drawing on and attempting to organize the reviewed evidence from fMRI, EEG, fNIRS, and ANS data focused specifically on altruistic motivation, aims to offer a thought-provoking structure that surpasses existing models predominantly based on single modalities or broader prosocial concepts, while clearly acknowledging its speculative nature and the need for extensive empirical validation. Based on the extensive body of prior research and the known functional architecture of brain networks implicated in social cognition, valuation, emotional processing, and cognitive control (Bellucci et al., 2020; Boccadoro et al., 2021; Correa et al., 2015; Cutler & Campbell-Meiklejohn, 2019; Filkowski et al., 2016; Hu et al., 2023), we anticipated that this scoping review would map distinct patterns of neurophysiological activity. Specifically, we expected to identify consistent engagement of brain regions critical for understanding others' mental states and intentions (e.g., temporoparietal junction (TPJ), medial prefrontal cortex (mPFC)), computing the subjective value of actions and their outcomes for self and other (e.g., ventromedial prefrontal cortex (vmPFC), striatum), processing and regulating emotions, particularly empathy (e.g., insula, anterior cingulate cortex (ACC)), and exerting cognitive control to overcome selfish impulses (e.g., dorsolateral prefrontal cortex (dlPFC)). We further anticipated that these central neural processes would be accompanied by discernible patterns of autonomic nervous system activity reflecting affective engagement, regulatory effort, or motivational states conducive to altruism, providing a more embodied and comprehensive neurobiological account.

We explicitly focus on costly, other-oriented altruism as operationalized in neuroimaging and psychophysiological literature, noting that included paradigms span both empathy-driven and fairness-based approaches. However, a systematic comparison of these subtypes or broader prosocial constructs lies beyond our current scope, and is highlighted as a limitation and direction for future meta-analyses.

## Methods

This scoping review was conducted and reported in accordance with the Preferred Reporting Items for Systematic Reviews and Meta-Analyses Extension for Scoping Reviews (PRISMA-ScR) checklist (Tricco et al., 2018). The primary objective was to systematically map and synthesize the existing literature on neurophysiological correlates (i.e., brain activity and autonomic nervous system responses) of our precisely defined construct of “altruistic motivation” in humans. Specifically, this review aimed to identify the key brain regions, neural networks, temporal dynamics, and physiological patterns consistently associated with intrinsically other-oriented, costly altruistic decision making and behavior, thereby providing a comprehensive and critically integrated overview of the current state of knowledge in this domain.

### Eligibility criteria

We synthesized only studies reporting state-dependent neurophysiological activity (fMRI, EEG, fNIRS, ANS, etc.) elicited during specific task-based, or intervention protocols. For clarity, “task-based” refers exclusively to participant engagement in a structured experimental paradigm intended to elicit altruistic motivation or behavior, and does not necessarily indicate that neurophysiological measurements were recorded online or concurrently with the task. Trait-level measures (including anatomical/structural, resting-state, and observational designs) were excluded. The absence of tDCS, neurofeedback, or trait-level studies in our review reflects the outcome of our systematic, protocol-based literature search, rather than any conceptual exclusion criterion.

Studies were included if they met the following criteria, structured around the Population, Concept, and Context (PCC) framework, along with study design considerations:

#### Participants

Studies involving human participants of any age or demographic background were eligible. While the initial search was broad, the synthesis primarily focuses on studies with healthy adult populations, though research including adolescents or specific altruistic groups (extraordinary altruists, individuals undergoing intervention) was also incorporated if other criteria were met. Studies exclusively focused on animal models were excluded. Studies comparing clinical populations to healthy controls regarding the neural basis of altruism were considered, though the final included set predominantly featured non-clinical samples or focused on altruism as a primary variable rather than a group difference marker linked to a disorder.

#### Concept

The review focused on studies that explicitly investigated the relationship between “altruistic motivation” – defined consistently with our Introduction as “the underlying psychological impetus to increase another’s welfare, where the ensuing behavior often involves some tangible cost (monetary, time, physical effort, social risk) to the actor” – and direct measures of neurophysiological activity. This included measures of brain function (fMRI, EEG, MEG, fNIRS, PET) and/or ANS activity (ECG, heart rate, heart rate variability, electrodermal activity). Studies where altruism was not a primary outcome or focus, or those relying solely on behavioral or self-report measures of altruism without accompanying neurophysiological data, were excluded. To systematically address the notable heterogeneity in how altruism is operationalized across studies, we charted two key variables for each included article: the specific “Cost Type” (e.g., monetary, effort, social risk) and a “Paradigm Classification” distinguishing “Core Altruistic” tasks from “Borderline/Strategic” ones. This classification, detailed in Table 1, informs our narrative synthesis and allows for a more nuanced interpretation of the evidence. We also systematically recorded whether paradigms provided a clear, personal, and other-oriented cost that aligns with our review’s operational definition, or if they used broader prosocial/reward contrasts.

**Table 1:** Characteristics of Included Studies (N = 45)

Author(s)	Year	Physiological measurement index	Specific task/paradigm	Design	Paradigm classification	Cost type
Eisenberg et al	1989	Heart rate	Exposure to a needy other (easy escape condition) with the choice to help or not	Experimental	Core altruistic	Time; Physical Effort
Rilling et al	2002	fMRI	Iterated Prisoner's Dilemma Game (cooperation and non-cooperation in social and non-social contexts)	Experimental	Borderline/strategic	Monetary
Moll et al	2006	fMRI	Charitable Donation Task (deciding to donate to or oppose real charitable organizations)	Experimental	Core altruistic	Monetary
Harbaugh et al	2007	fMRI	Financial transfers affecting own account and a charity's account (half mandatory, half voluntary)	Experimental	Core altruistic	Monetary
Hare et al	2010	fMRI	Charitable Donation Decisions (value-based, voluntary donation and compulsory donation)	Experimental	Core altruistic	Monetary
Caspers et al	2011	fMRI	Forced-choice paradigm on word pairs representing abstract values (collectivistic/altruistic vs. individualistic/egocentric)	Experimental	Core altruistic	Not clearly specified
Huffmeijer et al	2012	EEG (Frontal Asymmetry)	Donation Decision (after watching a promotional video about children in need)	Experimental	Core altruistic	Monetary
Kuss et al	2013	fMRI	Charitable Donation Task (decision on how to allocate money between you and charities)	Experimental	Core altruistic	Monetary
Weng et al	2013	fMRI	Compassion Training Intervention, Redistribution Game (images of human suffering or non-suffering)	Randomized Controlled Trial	Core altruistic	Monetary
Greening et al	2014	fMRI	Financial Helping vs Harming Decisions	Experimental	Core altruistic	Monetary
Rilling et al	2014	fMRI	Prisoner's Dilemma Game (intranasal administration effect)	Experimental	Borderline/strategic	Monetary
Zaki et al	2014	fMRI	modified dictator game (allocate money to yourself or to another participant)	Experimental	Core altruistic	Monetary
Zanon et al	2014	fMRI	Simulated Life-Threatening Situation (prosocial choice)	Experimental	Core altruistic	Physical Effort; Social Risk
Correa et al	2015	EEG (Theta, Mu), Heart Rate, HRV	Narrative Experience (reading stories), Donation Decision	Experimental	Core altruistic	Monetary
Haas et al	2015	fMRI	Empathic Accuracy Task, Emotional Perspective Taking Task (judging emotional facial expression matches in the social scene)	Experimental	Core altruistic	Not clearly specified
Himichi and Nomura	2015	fNIRS	Empathy Task (observing a card game) and Money Allocation Task (participants allocate money to subjects)	Experimental	Core altruistic	Monetary
Hu et al	2015	fMRI	Dictator Game (after observing an unfair situation, decide whether to punish or help at your own expense)	Experimental	Core altruistic	Monetary
Hutcherson et al	2015	fMRI	Modified Dictator Game (conditions for obtaining benefits for oneself and conditions for obtaining benefits for others)	Experimental	Core altruistic	Monetary
Rodrigues et al	2015	EEG (Theta)	Dictator Game (Fairness perception)	Experimental	Core altruistic	Monetary
Sul et al	2015	fMRI	Prosocial Choice Task (self vs. other monetary gain)	Experimental	Core altruistic	Monetary
Tamir et al	2015	fMRI	informing task, outcome observation task, and Monetary incentive delay task (reward and neutral trial)	Experimental	Core altruistic	Monetary
Hubbard et al	2016	fMRI	Donation Decisions, Self-Report Benevolence Scales	Experimental/Correlational	Core altruistic	Monetary
Kirk et al	2016	fMRI	Mindfulness Training Intervention, Anonymous version of the Ultimatum Game	Randomized Controlled Trial	Borderline/strategic	Monetary
Lockwood et al	2016	fMRI	Prosocial Learning Task (learning to benefit self vs other)	Experimental	Core altruistic	Monetary
Tusche et al	2016	fMRI	Charitable Donation Task (empathy/perspective-taking manipulation)	Experimental	Core altruistic	Monetary
Karns et al	2017	fMRI	Gratitude Intervention, Charitable Donation Task	Longitudinal (Intervention)	Core altruistic	Monetary
Oyediran and Rivas	2017	Heart Rate	dictator game (altruism and selfishness)	Experimental	Core altruistic	Monetary
Tousignant et al	2018	fMRI	Cyberball-tossing game (observing social exclusion)	Experimental	Core altruistic	Social Risk (observed exclusion)
Luo et al	2019	EEG (ERPs: FRN, P2, N2, fb-P3)	Monetary incentive delay task (hedonic vs. eudaimonic rewards)	Experimental	Core altruistic	Monetary
Piva et al	2019	fMRI	intertemporal choice task (self vs. other)	Experimental	Core altruistic	Monetary; Time
Liu et al	2020	EEG (ERPs: FRN, P300)	gambling game (self, high-empathy, and low-empathy condition)	Experimental	Borderline/Strategic	Monetary
Fede et al	2021	fMRI	Charitable Donation Task (charity preference, Perceived impact)	Experimental	Core altruistic	Monetary
Hu et al	2021	fMRI	interpersonal helping task (cost–benefit integration)	Experimental	Borderline/Strategic	Monetary; Physical Effort
Huang et al	2021	EEG (Multivariate Pattern Analysis)	The charitable donation task (positive, neutral or negative)	Experimental	Core altruistic	Monetary
Sellitto et al	2021	fMRI	Social Discounting Task (gain frame and loss frame)	Experimental	Borderline/Strategic	Monetary
Gan et al	2022	EEG (ERPs: FRN, P300)	Decision to donate after reading the story (support or not)	Experimental	Core altruistic	Monetary
Luo et al	2022	EEG (ERPs: RewP, P300)	revised monetary gambling task (hedonic vs. eudaimonic rewards)	Experimental	Borderline/Strategic	Monetary
Schulreich et al	2022	fMRI, Cortisol	Charitable Giving Task (stress induction, mentalizing capacity)	Experimental	Core altruistic	Monetary
Rhoads et al	2023	fMRI	Extraordinary Altruists (kidney donors) vs. Controls, Donation Task	Cross-Sectional	Core altruistic	Monetary; Physical (Kidney Donor)
Wijaya et al	2023	fMRI	Helping Decision Task (empathy dilemma, economic-dilemma)	Experimental/Correlational	Core altruistic	Monetary; Social Risk
Schulreich et al	2023	fMRI	Charitable donation task (decide how much to donate to the each charity)	Experimental	Core altruistic	Monetary
Kwon et al	2023	fMRI	Charity game (donating time or money)	Experimental	Core altruistic	Time; Monetary
Bas et al	2023	fMRI	Social perception task, altruism task (merit/need perception)	Experimental	Borderline/Strategic	Not clearly specified
Mao et al	2024	EEG (ERPs: N2)	Charitable donation task, effort-expenditure rewards task (self-interest and donation behavior)	Experimental	Core altruistic	Monetary; Physical Effort
Mitiureva et al	2024	EEG (weighted phase lag index)	Pain versus Gain task (altruistic decision making)	Experimental	Core altruistic	Monetary; Social Risk

To maximize conceptual precision, this review strictly defines “altruism” as behavior that incurs a tangible cost to the actor and is principally motivated by the intent to benefit another. We distinguished this from broader prosocial constructs, which may include helping, sharing, or cooperation when (a) the motivation is ambiguous or strategic (e.g., reciprocity, compliance, reputational benefits), or (b) no clear personal cost is present.

Accordingly, studies were excluded if they:

- Focused solely on empathy or observation of others’ pain, without requiring the participant to incur a cost.
- Examined fairness, in-group/out-group preferences, or general cooperation where other-oriented motivation or tangible cost was not explicit.
- Were primarily trait-level measures (including anatomical/structural, resting-state, and observational designs).
- Were reviews, meta-analyses, or commentary.

These decision rules are now detailed in the Online Supplementary (OSM) Material, Table S1, which enumerates representative excluded studies and rationales. This approach enhances transparency and supports reproducibility.

#### Context

No restrictions were placed on the geographical context or setting (laboratory, naturalistic), provided the study reported empirical neurophysiological data. Only articles published in English were included.

#### Study types

Eligible study designs included experimental studies, randomized controlled trials (RCTs), prospective longitudinal studies, and cross-sectional studies (including surveys with neurophysiological components). This breadth was chosen to capture the diverse methodologies employed in this research area. Review articles, meta-analyses, commentaries, letters, and case reports lacking original data on a sample of participants were excluded. Our protocol included studies employing fMRI, EEG, ANS, and fNIRS. However, only one fNIRS study met criteria; we flag this throughout as preliminary. Definitions of “altruism” and “cost” are variable across studies, as detailed in Table 1 and recorded for each included article.

### Information sources and search strategy

A systematic literature search, focused on identifying studies directly investigating the interplay of neurophysiology, altruism as defined, and decision making/motivation, was conducted across five electronic databases: PubMed, Scopus, CINAHL, The Cochrane Central Register of Controlled Trials (CENTRAL), and MEDLINE. The search encompassed articles published from 1 January 1975 to 31 December 2024, the date the search was completed. This broad timeframe was chosen to ensure comprehensive coverage of the development of neurophysiological research in altruism. Only peer-reviewed journal articles were considered for inclusion. Studies retrieved through broader search terms (e.g., “goodwill,” “magnanimity”) were carefully evaluated during the screening process to ensure their operationalization of the construct aligned with the review’s specific definition of altruistic motivation. Citations to studies published after 31 December 2024 are provided solely for contextual or illustrative purposes and do not contribute to the empirical syntheses of this review.

The search strategy was developed by two authors (J.O. and T.I.) and combined keywords and controlled vocabulary related to three core concepts: (1) neurophysiological measurement techniques, (2) altruism and constructs potentially reflecting altruistic motivation, and (3) motivation and decision-making process crucial for discerning genuine altruism. The exact search string employed was:

(EEG OR electroencephalography OR electroencephalogram OR MRI OR “Magnetic Resonance Imaging” OR fMRI OR MEG OR magnetoencephalography OR magnetoencephalogram OR NIRS OR “Near InfraRed Spectroscopy” OR PET OR “Positron Emission Tomography” OR SPECT OR “Single Photon Emission Computed Tomography” OR DTI OR “Diffusion Tensor Image” OR neurophysiology OR ANS OR “Autonomic Nerve” OR “autonomic nervous” OR ECG OR electrocardiogram OR electrocardiography OR “heart rate”)

AND (altruism OR charity OR goodwill OR magnanimity)

AND (“decision making” OR motivation OR “self-determination”).

The requirement for all three concept categories (neurophysiology AND altruism AND decision making/motivation) to be present in the search string was a deliberate choice to specifically target studies investigating the neural correlates of altruistic motivation leading to a decision, rather than broader prosocial behaviors or altruistic traits without an explicit decision-making component. While this focused approach aimed to increase specificity in line with our definition of altruistic motivation, we acknowledge that it may have led to a lower initial record count (N = 462) compared to broader searches on altruism and neurophysiology, and potentially excluded studies investigating neural correlates of altruistic acts without explicitly framing them within a decision-making or motivational paradigm. This specificity, however, was deemed critical for the aims of this particular scoping review.

The inclusion of terms like “goodwill” or “magnanimity” was strategic to cast a wide net initially; however, studies retrieved through these broader terms underwent rigorous screening (see *Study selection*) to ensure their operationalization of the construct and the presence of experimental conditions strictly aligned with this review’s specific definition of “altruistic motivation,” particularly concerning the actor’s intent and incurred cost. The “decision making,” “motivation,” and “self-determination” terms were included to help capture studies that investigated the underlying psychological impetus and volitional aspect of altruistic acts.

### Study selection

All records identified through the database searches were imported into Microsoft Excel for duplicate removal and screening. Two reviewers (J.O. and T.I.) independently screened titles and abstracts against the predefined eligibility criteria. Any discrepancies at this stage were resolved through discussion to reach a consensus. Full texts of potentially relevant articles were then retrieved and independently assessed for eligibility by the same two reviewers. Disagreements regarding full-text inclusion were resolved through discussion and, if consensus could not be reached, by consultation with a third reviewer (K.S.), a clinical expert with experience in neuroimaging and behavioral research. This consensus process ensured consistent application of eligibility criteria throughout the selection phase. The study selection process, including the number of records identified, screened, assessed for eligibility, and ultimately included in the review, is documented in a PRISMA flow diagram.

### Data-charting process

A data-charting form was developed collaboratively by the reviewers using Microsoft Excel, guided by the PRISMA-ScR recommendations. The two reviewers (J.O. and T.I.) independently extracted data from each included study and iteratively refined the form throughout the process to ensure comprehensive capture of relevant information. Extracted variables included:

- Bibliographic details (author(s), year of publication, title)
- Study objectives/aims
- Study design
- Participant characteristics (sample size, age, sex, population type – e.g., healthy adults, adolescents)
- Altruism operationalization (e.g., task paradigm used to elicit altruistic motivation/behavior, such as Dictator Game, charitable donation task, costly helping scenario)
- Comparison conditions or control tasks
- Neurophysiological methods employed
- Key neurophysiological findings related to altruistic motivation or behavior
- Reported limitations of the study.

Consistent with the objectives of a scoping review, a formal assessment of the methodological quality or risk of bias of individual studies was not performed. However, key aspects of study design were charted to provide context for the synthesis of findings.

During data charting, studies using strategic or potentially mixed-motive paradigms (e.g., Prisoner’s Dilemma, Public Goods Game) were flagged and, unless paradigm design strongly minimized strategic motives (i.e., one-shot, anonymous decisions), were considered “borderline.”

### Classification of altruistic paradigms

To ensure transparency and consistency, we classified experimental paradigms as “core altruistic” if both an explicit cost to the actor and a direct, identifiable benefit to another were present (e.g., Dictator Game, Charitable Donation tasks). Paradigms were classified as “borderline/strategic” if the cost to self or the benefit to others was ambiguous, not explicitly specified, or where strategic motives (e.g., anticipated reciprocity, repeated interactions) could not be ruled out (e.g., Prisoner’s Dilemma, Public Goods Game). Ambiguous cases were conservatively categorized as “borderline/strategic.” These criteria are systematically applied and explicitly flagged in Table 1.

### Data synthesis

The extracted data were synthesized narratively and presented in tabular format to provide an overview of the research landscape. Specifically, Table 1 summarizes the characteristics of the included studies (N = 45), including their design, participant samples, altruism paradigms, and neurophysiological techniques. Table 2 details the specific neurophysiological findings associated with altruistic motivation, categorized by measurement modality (fMRI, EEG, fNIRS, ANS) and, where applicable, by specific brain regions or physiological parameters. The synthesis aimed to identify common themes, patterns of neurophysiological correlates across studies, and gaps in the existing literature. The narrative synthesis focused on identifying recurrent findings and prominent trends rather than quantitatively pooling results, acknowledging the heterogeneity inherent in the field. The results were organized to address the review’s objective of clarifying the neural and physiological bases of altruistic motivation. As this study was designed as a scoping review, its primary aim was to map the breadth and nature of the existing literature on the neurophysiological correlates of altruistic motivation, rather than to conduct a quantitative statistical synthesis of study results, such as a meta-analysis. Therefore, quantitative meta-analysis, including the calculation of summary effect sizes, confidence intervals, or formal statistical measures of heterogeneity, was not planned or conducted. This decision was based on the methodological framework of scoping reviews, which typically prioritize mapping the literature over statistical aggregation, and also due to the anticipated significant heterogeneity in study methodologies, operationalizations of altruism, and outcome measures across the included studies. Similarly, a formal quantitative assessment of publication bias was not undertaken as part of this scoping review methodology.

**Table 2:** Results of a review of neurophysiological characteristics associated with altruism

Category	Neurophysiological indicator	Strengthens/enhances when altruistic	Weakened/decreased when altruistic	Strengthened by both altruism/selfishness	Strengthens/enhances when selfish
EEG	Left frontal activity (alpha asymmetry)	Huffmeijer et al., 2012		Correa et al., 2015	
	Middle frontal theta	Rodrigues et al., 2015		Correa et al., 2015	Rodrigues et al., 2015
	ERP: Frontal-central (180–440 ms)	Huang et al., 2021			
	ERP: Central-posterior (480–560 ms)	Huang et al., 2021			
	ERP: Frontal-medial and central-occipital (800–920 ms)	Huang et al., 2021			
	ERP: fb-P3 (Feedback P3)	Luo et al., 2019			
	ERP: P300	Gan et al., 2022			cue-P3—Luo et al., 2019 Liu et al., 2020
	ERP: N2	Mao et al., 2024		Luo et al., 2019	
	ERP: FRN		Gan et al., 2022	Liu et al., 2020	Luo et al., 2019
	ERP: RewP	Luo et al., 2022			
	Connectivity	rostral ACC and orbIFG in left hemisphere; right hemisphere network (FFG, PG, bSTS, MTG, Insula, cACC)—Mitiureva et al., 2024			
fMRI	Prefrontal				
	mPFC	Haas et al., 2015; Hubbard et al., 2016; Kwon et al., 2023; Rilling et al., 2002; Tousignant et al., 2018; Tusche et al., 2016			
	vmPFC	Hare et al., 2010; Karns et al., 2017; Kwon et al., 2023; Piva et al., 2019; Sellitto et al., 2021; Sul et al., 2015; Tamir et al., 2015		Hutcherson et al., 2015; Zaki et al., 2014	
	dmPFC	Kwon et al., 2023; Schulreich et al., 2022; Tousignant et al., 2018		Piva et al., 2019	Sul et al., 2015
	dlPFC	Hu et al., 2021; Hu et al., 2015; Kwon et al., 2023; Tusche et al., 2016; Weng et al., 2013			Lockwood et al., 2016
	vlPFC	Kwon et al., 2023			
	IFG	Sul et al., 2015			
	(right)	right—Fede et al., 2021			
	Middle frontal gyrus (right)	Caspers et al., 2011; Wijaya et al., 2023			
	Superior frontal gyrus (left)	Rhoads et al., 2023			
	Temporal				
	TPJ	Haas et al., 2015; Kwon et al., 2023; Schulreich et al., 2022			
	(right)	Bas et al., 2023; Greening et al., 2014; Hutcherson et al., 2015; Piva et al., 2019; Schulreich et al., 2022, 2023; Sellitto et al., 2021; Tousignant et al., 2018			
	pSTS	Hare et al., 2010; Tusche et al., 2016			
	Insular/cingulate/orbitofrontal				
	Insula	Harbaugh et al., 2007; Sellitto et al., 2021; Tusche et al., 2016 Male—Rilling et al., 2014	Kirk et al., 2016		
	(anterior)	Greening et al., 2014; Hu et al., 2021; Sul et al., 2015			
	(ventral)	Hu et al., 2021			
	PCC	Fede et al., 2021; Wijaya et al., 2023	Female—Rilling et al., 2014		
	ACC	Greening et al., 2014; Hu et al., 2021; Zanon et al., 2014			
	(rostral)	Hubbard et al., 2016; Rilling et al., 2002 More uniform mean activity in altruists—left—Rhoads et al., 2023			
	(dACC)	Kwon et al., 2023			
	(sgACC)	Lockwood et al., 2016			
	Middle cingulate cortex	Caspers et al., 2011			
	OFC	Rilling et al., 2002			
	Parietal				
	Inferior parietal cortex	Hu et al., 2021; Hu et al., 2015; Weng et al., 2013			
	(left rostral)	Caspers et al., 2011			
	Superior parietal cortex	Fede et al., 2021			
	Precuneus	Hutcherson et al., 2015; Kwon et al., 2023; Schulreich et al., 2022 More uniform mean activity in altruists—left—Rhoads et al., 2023			
	Occipital				
	Inferior occipital gyrus (right)	More uniform activity in altruists—Rhoads et al., 2023			
	Midbrain				
	VTA			Moll et al., 2006	
	Subcortical				
	Nacc	Hubbard et al., 2016; Karns et al., 2017; Rilling et al., 2002		Kuss et al., 2013	
	(left)	Tamir et al., 2015			
	(right)	Harbaugh et al., 2007			
	Striatum	Harbaugh et al., 2007; Hu et al., 2015; Moll et al., 2006; Rilling et al., 2002 Male—Rilling et al., 2014		Moll et al., 2006	
	(ventral)	Hubbard et al., 2016		Lockwood et al., 2016	Hutcherson et al., 2015
	Subfrontal areas (inc. BA25)	Moll et al., 2006			
	Amygdala (left)	Male—Rilling et al., 2014	Female—Rilling et al., 2014	Rhoads et al., 2023	Caspers et al., 2011
	Amygdala (right)	More uniform mean activity in altruists—Rhoads et al., 2023			
	Connectivity				
	mPFC subregions and striatum	Sul et al., 2015	Sul et al., 2015		
	Insula and cingulate cortex				Zanon et al., 2014
	mPFC and TPJ	Zanon et al., 2014			
	dlPFC and NAcc	Weng et al., 2013			
	Septal regions and posterior insular Cortex	Kirk et al., 2016			
	vmPFC and anterior insula	Hare et al., 2010			
fNIRS	vlPFC (left)	Himichi & Nomura, 2015			
HR/HRV	HR	Oyediran & Rivas, 2017	Eisenberg et al., 1989	Correa et al., 2015	
	HRV LF:HF ratio		Correa et al., 2015		

ERP: event-related potential; FRN: feedback-related negativity; RewP: reward positivity; mPFC: medial prefrontal cortex; vmPFC: ventromedial prefrontal cortex; dmPFC: dorsomedial prefrontal cortex; dlPFC: dorsolateral prefrontal cortex; vlPFC: ventrolateral prefrontal cortex; IFG: inferior frontal gyrus; TPJ: temporoparietal junction; pSTS: posterior superior temporal sulcus; PCC: posterior cingulate cortex; ACC: anterior cingulate cortex; sgACC: subgenual ACC; OFC: orbitofrontal cortex; VTA: ventral tegmental area; NAcc: nucleus accumbens; BA: Brodmann area; HRV: heart-rate variability; HR: heart rate

## Results

### Study selection

**Fig. 1:**
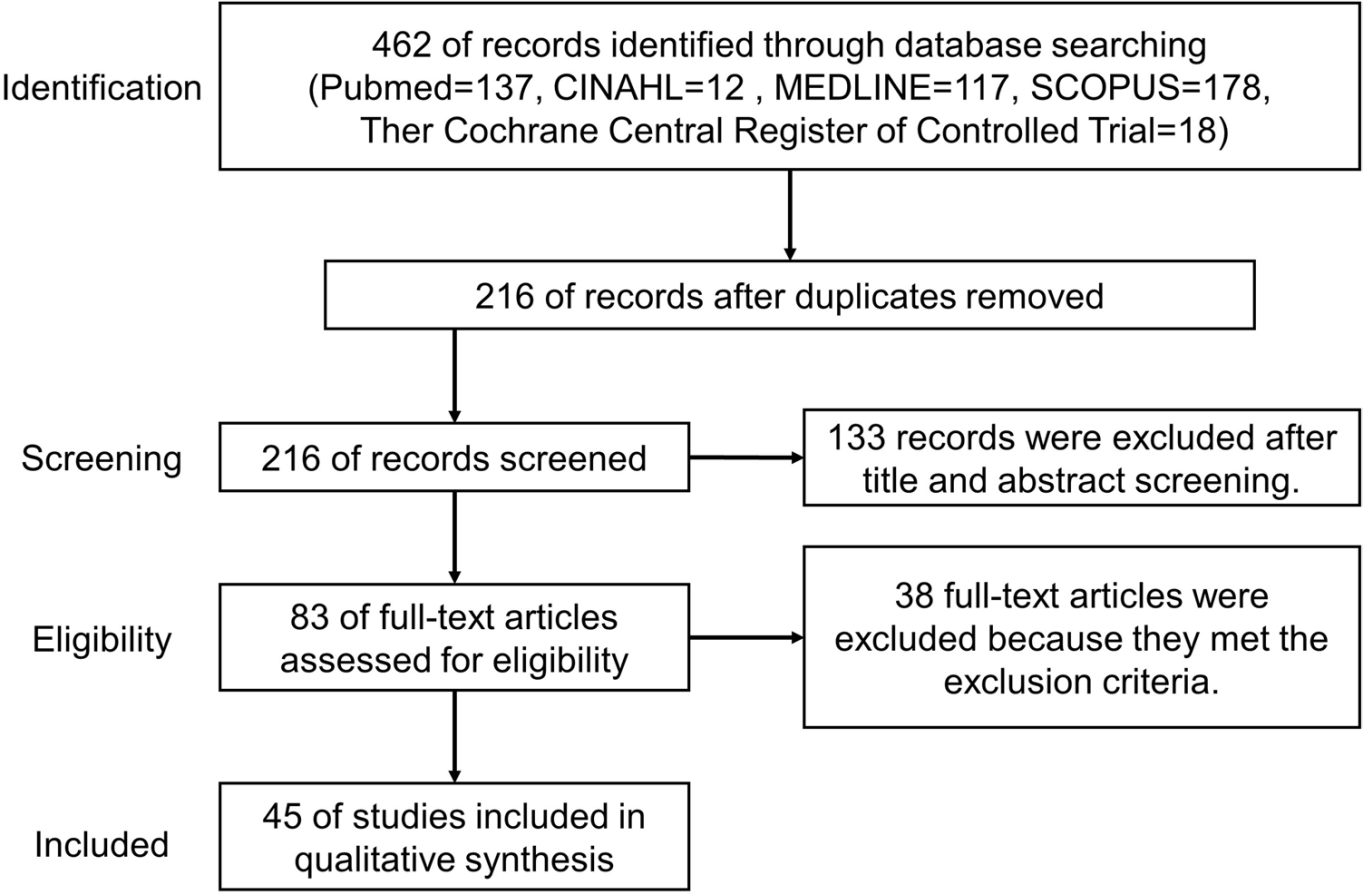
PRISMA flow diagram illustrating the study selection process

A database search initially yielded 462 records. After a rigorous screening process that included the removal of duplicates, 83 full-text articles were assessed for eligibility. A final set of 45 studies met the inclusion criteria and were included in this review. The study selection process is detailed in the PRISMA flow diagram (Fig. 1). To further clarify our operational criteria, we provide a summary table (OSM Table S1) of studies excluded at full-text screening. These predominantly failed to meet our requirements for both tangible personal cost and explicit other-orientation. Examples include paradigms limited to empathy/pain observation, studies centered on fairness or group loyalty, and tasks lacking robust altruistic motivation. This resource illustrates the rigorous boundaries applied in differentiating altruism from broader prosociality.

### Study characteristics

The final sample of 45 studies comprised a variety of research designs: one cross-sectional survey, 41 experimental studies, one longitudinal study, and two RCTs. The study populations primarily consisted of healthy adults. However, some studies focused on specific populations, such as adolescents, individuals characterized by high levels of altruism, or participants in intervention programs. The specific measures, tasks, and designs employed in each study are summarized in Table 1, while the neurophysiological characteristics associated with altruism are presented in Table 2.

### Experimental paradigms for eliciting altruism

As detailed in Table 1, the included studies utilized a range of experimental paradigms to elicit altruistic motivation. Game-theoretic paradigms were frequently employed (N = 13), with the Dictator Game, Prisoner’s Dilemma Game, and Ultimatum Game being the most common. These paradigms typically involved participants making choices between selfish monetary outcomes and costly other-benefiting monetary outcomes, thereby directly probing the willingness to incur personal cost for another’s gain. Donation and charity tasks were also frequently used (N = 11), typically requiring participants to decide how much of an endowment to keep versus donate. Other tasks included specific decision-making scenarios, tasks focusing on empathy and social cognition, and miscellaneous paradigms. Altruism was primarily operationalized as costly helping or resource allocation that benefited others. As detailed in Table 1, the included studies vary significantly in their experimental paradigms and definitions of “cost.” Our synthesis therefore distinguishes between findings from “Core Altruistic” paradigms, which offer the most direct evidence for our definition of altruism, and those from ‘Borderline/Strategic’ paradigms, which are interpreted with greater caution. Notably, the operationalization of “cost” varied across studies, with some using monetary costs, others physical effort, and still others social risk. This variability is a characteristic of the field and should be considered when interpreting the findings.

We carefully classified studies as follows:

- “Core altruistic” paradigms (e.g., Dictator Game, charitable donation tasks) directly operationalize costly other-oriented helping under conditions that minimize the likelihood of strategic or reciprocal motivation (e.g., one-shot, anonymity).
- “Borderline/strategic” paradigms (e.g., Prisoner’s Dilemma, Public Goods Game, repeated interactions) comprise settings where prosocial behavior may be influenced by anticipated reciprocal gain, reputation, or joint payoff maximization, and thus do not meet strict psychological/altruistic criteria. These paradigms are marked accordingly in Table 1, and their results are interpreted with caution and analytic separation.

In keeping with our strict definition, we created a separate synthesis for studies using the Prisoner’s Dilemma and other cooperation paradigms where the underlying motivation may include substantial strategic elements. Many prisoner’s dilemma and cooperation tasks permit prosocial choices motivated by anticipation of future reciprocal benefit, concern for reputation, or group payoffs – all of which can be considered strategic rather than intrinsically altruistic. Nevertheless, we include such studies for several reasons:

- They are prevalent in the literature mapping the neurophysiology of prosociality, and their neural findings often overlap with “core” altruistic paradigms.
- Some variants minimize strategic motives (e.g., one-shot, anonymous games); in these cases, prosocial responses may more closely reflect altruistic motivation.
- Including and explicitly flagging them enables a more nuanced mapping of the field and facilitates distinctions between intrinsic and strategic prosociality at the neural level.
- However, we do not interpret their results as direct evidence for altruistic motivation per se, and instead regard them as illuminating the broader neurobiology of prosocial choice, with carefully stated interpretive boundaries.

### Neurophysiological assessment techniques

The reviewed studies employed various neurophysiological methods (Table 1). fMRI was the most prevalent technique (N = 32), offering high spatial resolution to identify involved brain regions. EEG was used in ten studies, providing high temporal resolution insights into the dynamics of neural processing, with five studies focusing on event-related potentials and three on frequency band analysis. ANS activity, primarily heart rate or heart rate variability, was the main focus in three studies and included as a secondary measure in others. Only one study utilizing fNIRS met criteria; therefore, findings regarding this modality are highly tentative and should be interpreted as forward-looking rather than definitive. This distribution reflects the current landscape of neurophysiological research into altruism and allows for a broad, albeit heavily weighted by fMRI studies, mapping of findings. This combination of techniques allowed for a potentially comprehensive investigation of the neural and physiological underpinnings of altruism, capturing both spatial and temporal aspects of brain activity, as well as peripheral physiological responses potentially indicative of the affective and motivational states central to altruism, though the evidence base for each modality varies considerably.

### fMRI findings

fMRI studies, the majority of which employed “Core Altruistic” paradigms with tangible monetary or effort-based costs (see Table 1), consistently revealed the engagement of a distributed brain network during altruistic decision making and behavior, rather than a single localized region. As summarized in Table 2, key findings include:

#### Prefrontal cortex

Increased activation in various prefrontal regions was frequently observed. The mPFC, vmPFC, showed heightened activity during the evaluation and representation of subjective value associated with altruistic choices, such as charitable donations or cooperation (Haas et al., 2015; Hare et al., 2010; Hubbard et al., 2016; Karns et al., 2017; Kwon et al., 2023; Piva et al., 2019; Rilling et al., 2002; Sellitto et al., 2021; Sul et al., 2015; Tamir et al., 2015; Tousignant et al., 2018; Tusche et al., 2016). The dmPFC was also implicated, potentially in social decision making or conflict resolution (Kwon et al., 2023; Schulreich et al., 2022; Tousignant et al., 2018). Furthermore, the dlPFC exhibited increased activity during tasks requiring cognitive control to override selfish impulses or implement costly helping decisions (Hu et al., 2015, 2021; Kwon et al., 2023; Tusche et al., 2016; Weng et al., 2013). These findings suggest that the prefrontal cortex plays a crucial role in value assessment, social cognition, and cognitive control during altruistic behavior.

#### Temporoparietal regions

The TPJ, a region critical for social cognition processes such as perspective-taking and theory of mind, consistently showed greater activation during altruistic decisions, particularly when understanding others' intentions or mental states was relevant (Bas et al., 2023; Greening et al., 2014; Haas et al., 2015; Hutcherson et al., 2015; Kwon et al., 2023; Piva et al., 2019; Schulreich et al., 2022; Sellitto et al., 2021; Tousignant et al., 2018). Activity in the posterior superior temporal sulcus (pSTS) was also observed in related contexts (Hare et al., 2010; Tusche et al., 2016). Notably, Schulreich et al. (2023) found that higher subjective socioeconomic status was associated with stronger value coding in the right TPJ during charitable giving, suggesting a link between social status, mentalizing, and altruism. These findings highlight the importance of social cognitive processes in altruistic decision making.

#### Insula and cingulate cortex

The insula, particularly the anterior portion, was frequently activated, potentially reflecting empathic responses, processing of social-emotional salience, or interoceptive awareness related to altruistic feelings (Greening et al., 2014; Hu et al., 2021; Sul et al., 2015). The ACC showed involvement, possibly related to conflict monitoring (between self-interest and altruism), integrating emotional information, or encoding the effort/cost of helping (Greening et al., 2014; Hu et al., 2021; Hubbard et al., 2016; Kwon et al., 2023; Lockwood et al., 2016; Rhoads et al., 2023; Rilling et al., 2002; Zanon et al., 2014). The posterior cingulate cortex (PCC) was also implicated in some studies (Fede et al., 2021; Wijaya et al., 2023). These regions are thought to contribute to the emotional and motivational aspects of altruism.

#### Subcortical regions

Activation in reward-related regions, such as the striatum and nucleus accumbens (NAcc), was consistently linked to altruistic acts (Harbaugh et al., 2007; Hu et al., 2015; Hubbard et al., 2016; Karns et al., 2017; Moll et al., 2006; Rilling et al., 2002, 2014; Tamir et al., 2015). These findings align closely with affective neuroscience theories, most notably the “warm glow” hypothesis, which posits that prosocial acts elicit intrinsic hedonic rewards. The recruitment of this circuitry during charitable giving supports a model where moral actions and positive affect are tightly integrated at the neural level, suggesting that the drive to improve others’ welfare is reinforced by the brain’s fundamental motivational systems. The amygdala’s involvement was less consistent, though it was sometimes implicated in processing the emotional salience of altruistic scenarios or in relation to individual differences. The amygdala showed mixed results, sometimes implicated in processing the emotional significance of altruistic situations or correlating with individual differences (Rhoads et al., 2023). These findings suggest that altruistic behaviors can be intrinsically rewarding and are modulated by individual emotional responses.

#### Connectivity

Several studies emphasized the importance of functional interactions between brain regions. For example, connectivity between the dlPFC and NAcc predicted increases in altruism after compassion training (Weng et al., 2013), vmPFC-anterior insula connectivity was linked to value integration (Hare et al., 2010), and mPFC-TPJ interactions were observed during prosocial choices (Zanon et al., 2014). These connectivity findings emphasize that altruism relies on coordinated activity across networks involved in valuation, social cognition, emotion, and cognitive control. These findings highlight the importance of network-level interactions in supporting altruistic behavior.

### EEG findings

EEG studies provided valuable insights into the temporal dynamics of neural processes underlying altruism. However, EEG findings are predominantly based on paradigms where “cost” ranged from explicit monetary donation (e.g., Correa et al., 2015) to hedonic/eudaimonic reward-type contrasts (e.g., Luo et al., 2019) in which a direct cost to self is less evident. When discussing these EEG results, we explicitly note the paradigm and cost type involved for accurate interpretation.

#### Frequency bands

Increased relative left frontal activity (alpha asymmetry), potentially indicating approach motivation, was associated with altruistic tendencies or decisions in some contexts (Huffmeijer et al., 2012). Increased frontal midline theta (Fmθ) power, often linked to ACC activity and cognitive control or conflict monitoring, was observed during tasks requiring altruistic decisions or navigating fairness considerations (Correa et al., 2015; Rodrigues et al., 2015). These findings suggest that altruistic behavior is associated with specific patterns of brainwave activity related to motivation and cognitive control.

#### Event-related potentials (ERPs)

Different ERP components were modulated by altruistic contexts, suggesting influences at various processing stages. Multivariate analyses identified predictive patterns across several time windows (Huang et al., 2021). Later positive components like the P300 or feedback-P3 (fb-P3), often reflecting attentional allocation, context updating, or evaluation of outcome salience, were enhanced for altruistic outcomes compared to non-altruistic or hedonic ones (Gan et al., 2022; Luo et al., 2019). Conversely, the Feedback-Related Negativity (FRN), typically sensitive to unfavorable outcomes or prediction errors, was sometimes attenuated following altruistic outcomes, perhaps indicating reduced sensitivity to personal non-gain when benefiting others (Gan et al., 2022; Liu et al., 2020; Luo et al., 2019). Recent research by Mitiureva et al. (2024) further supports the role of EEG in understanding altruistic decision making by analyzing EEG-based functional connectivity patterns, which revealed that altruistic decisions correlate with emotional empathy and synchronous activity in the right hemisphere structures involved in empathy for pain. These ERP findings suggest that altruistic motivation influences not only late-stage evaluation but potentially also earlier attentional or valuation processes, consistent with computational modeling work suggesting early origins for altruistic biases. These findings provide evidence that altruistic motivation can affect early attentional and evaluative processes in the brain.

### ANS activity findings

Studies measuring autonomic activity yielded more mixed results, partly due to the small number of studies and the heterogeneity of paradigms, which ranged from classic costly helping tasks to more ambiguous scenarios. Given this variability, the specificity of any physiological signature for “costly” altruism remains tentative.

#### Heart rate (HR)

Findings were inconsistent. Some studies reported lower HR associated with sympathy or compassion (Eisenberg et al., 1989), while another found faster response times (potentially reflecting cognitive processing rather than state) accompanied by higher HR during moral dilemmas involving altruism (Oyediran & Rivas, 2017). The relationship between heart rate and altruism appears to be complex and context-dependent.

#### Heart-rate variability (HRV)

Some evidence suggested a link with parasympathetic activity. One study found a lower LF/HF ratio (indicating relative parasympathetic dominance) during narrative engagement preceding donations (Correa et al., 2015). This finding suggests that increased parasympathetic activity may be associated with altruistic states or dispositions.

#### Overall autonomic pattern

While tentative, some findings point towards increased parasympathetic influence or a state of physiological calm being associated with altruistic states or dispositions, potentially reflecting reduced stress or enhanced socio-emotional engagement. However, the inconsistency and the very small number of primary studies (N = 3) focusing on ANS activity as a main outcome highlights the need for more research carefully controlling for task demands, cognitive load, respiration, and individual differences. Further research is needed to clarify the relationship between ANS activity and altruistic behavior. The inclusion of even a small number of ANS and the single fNIRS study aims to provide a comprehensive map of all modalities currently used, highlighting areas ripe for future development. Therefore, claims regarding specific autonomic signatures of altruism based on the current evidence must be considered highly speculative and require substantial further investigation. While the inclusion of even a small number of ANS and the single fNIRS study aims to provide a map of all modalities currently used, this also highlights areas ripe for future development rather than established patterns for these less-represented modalities.

### Synthesis of findings across methods

Taken together, the findings from fMRI, EEG, and ANS studies suggest that altruistic motivation, defined by its other-oriented impetus and inherent cost to the actor, involves the interplay of multiple neural systems. fMRI studies have consistently implicated a distributed network encompassing prefrontal regions (mPFC, vmPFC, dlPFC), TPJ, and limbic/paralimbic structures (insula, ACC, striatum), highlighting the cognitive (value computation, perspective-taking, cognitive control) and affective (empathy, intrinsic reward) components. EEG studies complement these spatial insights by revealing temporal dynamics, indicating that relative increases in left frontal activity (approach motivation) and changes in frontal midline θ rhythm (cognitive control) are associated with altruistic tendencies and decision making, while ERPs track attentional allocation and outcome evaluation specific to altruistic contexts. Studies of ANS activity, though more varied, tentatively suggest that states of parasympathetic dominance may facilitate or accompany altruistic motivation, though this requires more robust investigation. Overall, these multi-modal findings support the notion that altruistic motivation arises from a complex interaction of cognitive and emotional processes, engaging a distributed network of brain regions and associated physiological systems, all orchestrated to enable costly actions aimed at enhancing another’s welfare.

## Discussion

This scoping review systematically mapped and synthesized recent research investigating the neurophysiological underpinnings of altruistic motivation in humans, drawing upon findings from fMRI, EEG, fNIRS, and ANS studies. The comprehensive analysis of 45 studies consistently demonstrates that altruism is not subserved by a single, isolated brain region or physiological process. Instead, it emerges from the complex, dynamic interplay of distributed neural networks and physiological systems involved in social cognition, value-based decision making, reward processing, cognitive control, and emotional responding. This synthesis specifically advances understanding beyond prior reviews by focusing meticulously on intrinsically motivated, costly altruism and by integrating evidence across these four neurophysiological modalities, offering a more granular and holistic perspective.

Notably, recent studies suggest that the type of altruistic cost, such as physical effort versus monetary sacrifice, may influence which neural circuits are engaged. For example, existing fMRI research indicates that while both cost types commonly activate core prosocial and valuation regions, including the mPFC, TPJ, and insula (Haas et al., 2015; Kwon et al., 2023; Sul et al., 2015), effort-based altruism more robustly recruits motor and premotor areas, whereas monetary giving preferentially engages reward-related regions such as the vmPFC and striatum (Harbaugh et al., 2007; Hare et al., 2010). However, direct, within-subject comparative research remains limited, and future studies employing explicit contrasts between cost domains are critically needed to clarify these modality-dependent effects.

Our findings underscore the need for rigorous distinction between altruistic and strategic prosocial motivation at both the behavioral and neurophysiological level. The present review is among the first to systematically flag and, where possible, analytically separate “core” altruistic paradigms from those involving strategic motives, including dedicated commentary and limitations for the latter. While neural networks implicated across paradigms show substantial overlap, it is crucial to note that in the Prisoner’s Dilemma and similar tasks, neural activation may not reflect “altruistic” motivation in the strict psychological sense, but rather the cognitive and affective mechanisms supporting broader prosocial orientation, reciprocity, or group-mindedness. This analytic approach helps clarify why some earlier neuroimaging meta-analyses yielded inconsistent or diffuse results for “altruism”: operational slippage between truly altruistic and strategic paradigms. Our findings argue for continued careful control, explicit reporting, and interpretive caution. A central challenge in synthesizing this literature is the marked heterogeneity in the operationalization of “cost.” While paradigms involving explicit monetary or effort-based costs – prevalent in the fMRI literature – provide direct evidence for our definition of altruistic motivation, other paradigms, particularly some in EEG research, feature less direct costs. Our analysis, which separates these paradigms (see Table 1), underscores that broad conclusions about a single “neurobiology of altruism” must be tempered. Future research requires greater harmonization of cost-related paradigms to move towards meta-analytic integration.

### Converging evidence for a distributed neural network underlying altruism

The findings from fMRI studies, which constituted the largest body of evidence in this review (N = 32), provide compelling support for a multi-component neural architecture of altruism. Key regions consistently implicated include the mPFC; particularly vmPFC, dlPFC, TPJ, insula, and ACC, PCC. The vmPFC’s role in computing the subjective value of altruistic actions, such as charitable donations or cooperation (Cutler & Campbell-Meiklejohn, 2019; Hare et al., 2010), appears central, often integrating social information and tracking the value assigned to outcomes for others (Piva et al., 2019). The observation of a potential functional gradient along the mPFC, with ventral regions more tied to value computation and dorsal regions to social decision making or conflict resolution (Sul et al., 2015), warrants further investigation into how these subregions distinctively contribute to complex altruistic choices.

The TPJ, a critical hub for social cognitive functions like perspective-taking and theory of mind (Van Overwalle, 2009), consistently showed heightened activation, particularly when understanding others’ intentions was paramount (Haas et al., 2015; Kwon et al., 2023). Notably, Schulreich et al. (2023) provided recent evidence linking higher subjective socioeconomic status to stronger value coding in the right TPJ during charitable giving, suggesting an intriguing modulation of mentalizing-related valuation by social contextual factors. This highlights the cognitive empathy component integral to many forms of altruism. Concurrently, the dlPFC’s involvement seems crucial for implementing altruistic intentions, potentially by exerting cognitive control to override prepotent selfish impulses or manage the costs associated with helping (Hu et al., 2021; Weng et al., 2013).

The insula, particularly its anterior division, was frequently implicated, likely reflecting the processing of empathic responses, interoceptive awareness of altruistic feelings, and the socio-emotional salience of the situation (Greening et al., 2014; Hu et al., 2021; Sul et al., 2015). The ACC’s engagement in conflict monitoring (e.g., between self-interest and other-regarding goals), integrating emotional and cognitive information, and potentially encoding the effort or cost of helping (Hu et al., 2021; Lockwood et al., 2016) further underscores the multifaceted nature of altruistic decision-making. The consistent co-activation of these regions, vital for higher-order social cognition, valuation, executive function, and emotion processing (Haber & Knutson, 2010; Phan et al., 2002; Van Overwalle, 2009), strongly supports the notion that altruistic motivation arises from the sophisticated orchestration of these diverse neural systems.

Beyond regional activations, this review highlighted the burgeoning field of inter-regional connectivity in understanding altruism. As noted in our *Results* section, studies have demonstrated functional coupling between regions like the dlPFC and NAcc (linking cognitive control to reward processing in compassion-trained altruism; Weng et al., 2013), vmPFC and anterior insula (integrating value and affective signals; Hare et al., 2010), and mPFC and TPJ (integrating value and social cognition during prosocial choices; Zanon et al., 2014). These findings underscore that altruistic motivation is not simply the sum of isolated regional activities but relies on the coordinated information flow across large-scale neural networks. This network perspective suggests a dynamic process where altruistic acts may involve evaluating potential benefits to others (TPJ, mPFC), integrating this with personal values and costs/rewards (vmPFC, striatum/NAcc), experiencing relevant emotions like empathy (insula, ACC), and executing the decision via cognitive control (dlPFC, ACC). This resonates with the idea that altruistic behavior can be intrinsically rewarding, a concept supported by observed “warm glow” activity in striatal regions (Harbaugh et al., 2007).

### Temporal dynamics and psychophysiological signatures: Insights from EEG and ANS

While fMRI provides unparalleled spatial resolution, EEG studies (N = 10), though fewer in number, offer invaluable high-temporal-resolution insights into the rapid neural dynamics underpinning the emergence and execution of altruistic cognition and motivation. Our review indicates that altruistic states and decisions are associated with distinct electrophysiological signatures. Notably, increased relative left frontal cortical activity, commonly indexed by frontal alpha asymmetry (FAA), has been linked to altruistic tendencies or choices (Huffmeijer et al., 2012). This FAA pattern is widely interpreted as reflecting heightened approach motivation (Coan & Allen, 2003; Harmon-Jones & Allen, 1997), suggesting that altruism, at least in some contexts, may be driven by an active motivational system geared towards engaging with and benefiting others, rather than merely by the avoidance of negative social consequences or guilt. However, its role in altruism remains under-explored. Given robust evidence from affective neuroscience linking left frontal activation to approach-related positive affect, its presence during altruistic acts may signal more than simple motivation; it may reflect the engagement of positive emotional systems that reinforce and sustain prosocial behavior. Future research should therefore test whether condition-specific lateralization patterns mediate the relationship between altruistic choice and subsequent self-reports of positive affect. Furthermore, enhanced Fmθ power, an oscillatory rhythm thought to emanate largely from the ACC and adjacent mPFC structures (Cavanagh et al., 2013; Ishii et al., 1999), was consistently observed during tasks requiring altruistic decisions or the navigation of fairness considerations (Correa et al., 2015; Rodrigues et al., 2015). This Fmθ activity likely reflects the engagement of crucial cognitive control processes, such as monitoring conflict between selfish desires and prosocial goals, overriding prepotent self-interest, sustaining attention on other-relevant information, or updating action plans based on social feedback.

ERP analyses further illuminate the stages of information processing influenced by altruistic motivation. Modulations of later positive components like the P300 or fb-P3 – often reflecting attentional allocation to motivationally salient stimuli, context updating, and the evaluative processing of outcome significance – were typically enhanced for altruistic outcomes compared to non-altruistic or purely hedonic ones (Gan et al., 2022; Luo et al., 2019). This suggests that outcomes benefiting others can capture greater attentional resources and undergo more elaborate evaluative processing, perhaps signifying their heightened subjective importance within an altruistic motivational framework. Conversely, the FRN, an ERP component sensitive to unfavorable outcomes or prediction errors, was sometimes attenuated following altruistic choices that did not yield personal gain (Gan et al., 2022; Luo et al., 2019). This intriguing attenuation might indicate a recalibration of the brain’s reward prediction error system, where the fulfillment of an altruistic goal (benefiting another) mitigates the “error” signal typically associated with the absence of personal reward, effectively prioritizing the other’s welfare in the neural valuation process. The recent sophisticated analysis of EEG-based functional connectivity patterns by Mitiureva et al. (2024), revealing correlations between altruistic decisions, emotional empathy, and synchronous activity in right hemisphere structures implicated in empathy for pain, provides compelling evidence for the dynamic neural interplay underlying the affective components of altruism, captured with millisecond precision. Collectively, these EEG findings powerfully suggest that altruistic motivation profoundly shapes neural information processing across multiple temporal stages, from early attentional capture and motivational orientation, through executive control engagement, to the ultimate evaluation of action outcomes.

Investigations of ANS activity present a more nuanced and, at times, less consistent picture, stemming from a considerably smaller evidence base (N = 3 primarily focused studies and some others with secondary measures) compared to central neural measures, yet offer potential insights into the embodied nature of altruism. Some studies have reported decreased HR in association with states of sympathy or compassion (Eisenberg et al., 1989), potentially reflecting a state of calm, other-focused attention conducive to prosocial behavior. Conversely, other research found increased HR in specific, often acute, moral dilemma contexts (Oyediran & Rivas, 2017), possibly indicating heightened arousal or cognitive effort. This variability in HR findings likely stems from several factors, including the heterogeneity of altruistic paradigms (e.g., passive observation vs. active decision making under pressure), differing emotional concomitants (e.g., empathic distress vs. empathic joy), uncontrolled cognitive load, and individual differences in baseline arousal or reactivity. HRV, particularly measures reflecting parasympathetic influence (high-frequency HRV, or a lower LF/HF ratio), has shown somewhat more consistent, albeit still tentative, links with altruistic tendencies. For instance, Correa et al. (2015) found a lower LF/HF ratio during narrative engagement preceding donations. Such findings hint at a potential role for increased parasympathetic activity – or a state of relative sympathetic quiescence – in fostering altruistic states or dispositions. This aligns with theories like Porges’ Polyvagal Theory (Porges, 2011), which posits that the ventral vagal complex supports states of safety, social connection, and calm engagement, all of which are theoretically conducive to altruistic behavior. A physiological state characterized by reduced sympathetic hyperarousal and enhanced parasympathetic tone might facilitate greater openness to others, reduced self-protective responses, and an enhanced capacity for empathic attunement. Investigations of ANS activity present a more nuanced and, at times, less consistent picture. For instance, some studies report heart rate deceleration in contexts of sympathy or compassion, while others find acceleration during high-stakes, costly helping decisions. These divergent findings likely arise from critical contextual moderators. Paradigms designed to elicit calm, affiliative states of compassion may promote parasympathetic dominance, whereas those involving acute, morally demanding choices may induce sympathetic arousal. This underscores the necessity for future studies to systematically manipulate and measure these contextual factors to clarify the physiological profiles accompanying distinct forms of altruistic motivation.

However, the current ANS literature on altruism is limited, and the interpretation of these findings must be particularly cautious due to methodological heterogeneity and the small number of studies. The claims about autonomic signatures of altruism are therefore highly tentative and require substantial further investigation. The limited number of dedicated ANS studies and the methodological heterogeneity underscore the critical need for more systematic research. It is important to note that while not falling within the publication timeframe or specific inclusion criteria of the studies formally synthesized in this review, emerging research exemplifies the potential of combining EEG and autonomic measures. For instance, a recent pilot study by Orui (2025), mentioned in the article, suggests that tasks motivated by altruistic intentions (implying benefit to other at cost to self) are associated with increased relative left frontal EEG activity (indicative of approach motivation) and concurrently reduced sympathetic activity (suggesting diminished hyperarousal or stress). Such multimodal findings, if replicated and extended with rigorous control over the operationalization of “cost” and “other-oriented impetus,” could offer a more integrated psychophysiological signature of truly altruistic states, potentially reconciling some of the current variability observed when ANS measures are considered in isolation and paving the way for a more holistic understanding of mind-body interactions in altruism. However, this is cited only for contextual illustration and not as part of the systematic review. Such recent findings exemplify the potential of combining EEG and autonomic measures, but are not part of the systematic evidence base. Although our systematic search included fNIRS, only a single such study met the present review’s criteria. As such, the discussion of fNIRS findings is limited, and any generalizations about its relevance to the neurobiology of altruistic motivation should be regarded as preliminary.

### Theoretical implications and a refined speculative model of altruistic processing

The collective findings from this review resonate strongly with, and provide neurobiological instantiation for, several prominent theoretical frameworks of social behavior. The consistent and pivotal engagement of the mPFC and TPJ lends robust neuroscientific support to social cognitive theories that emphasize the critical role of understanding others’ mental states in guiding adaptive social interactions (Bandura, 1986). From a decision neuroscience vantage point, the recruitment of the dlPFC, vmPFC, and ACC during altruistic choices highlights the complex interplay of valuation, cognitive control, and conflict resolution inherent in navigating decisions that often pit immediate self-interest against longer-term social goals or other-regarding values (Ruff & Fehr, 2014). Furthermore, the robust engagement of reward-related circuitry (e.g., striatum, NAcc, vmPFC) is consonant with evolutionary, psychological, and neuroeconomic theories positing that altruism, far from being purely self-sacrificial, can confer intrinsic hedonic rewards (the “warm glow”), fulfill fundamental human needs for social connection and competence, or align with deeply held moral values (Deci & Ryan, 1985; Sober & Wilson, 1998).

Our critical synthesis identifies key empirical gaps, such as the paucity of longitudinal and ecologically valid studies, and the limited integration of cross-modality data (e.g., concurrent fMRI-EEG-ANS measurements). Based on the model, we propose several testable predictions for future research: for instance, individual differences in left frontal alpha asymmetry during a donation task might predict the monetary value of subsequent altruistic giving. We hypothesize this relationship will be statistically mediated by self-reported “warm glow” or positive affect; or that task-induced context (e.g., empathy induction vs. moral dilemma) will differentially modulate the temporal coupling between prefrontal, parietal, and subcortical networks. Elucidation of these predictions will be critical for transforming the current heuristic model into an empirically grounded framework.

To conceptually organize the diverse neurophysiological evidence reviewed, we propose a multi-component heuristic framework. It is crucial to emphasize that this model is intended as a conceptual organizer to structure the complex findings, rather than a validated model of strictly sequential information processing. The primary utility of this framework lies in its capacity to map findings from different modalities onto potential processing components and to generate specific, testable hypotheses for future research. The constituent components are likely interactive and recurrent, and their precise temporal unfolding and causal interdependencies remain critical frontiers for empirical inquiry.

The following heuristic framework outlines potential processing components:

1. Socio-Affective Appraisal and Mental State Inference: This initial stage involves detecting and evaluating another’s needs or emotional state. This component is supported by a robust body of fMRI studies included in this review, which consistently demonstrate activation of the TPJ and pSTS in tasks requiring perspective-taking (e.g., Haas et al., 2015; Hare et al., 2010; Tusche et al., 2016). We explicitly place the TPJ here to reflect its critical role in understanding others' mental states, a point highlighted throughout this review.
2. Empathic Resonance and Affective Valuation: This component involves generating an empathic response and computing the subjective value of the altruistic act. This is supported by robust findings of activation in the anterior insula, ACC, and vmPFC (e.g., Greening et al., 2014; Sul et al., 2015). These regions are central to integrating emotional salience with value computation.
3. Motivational Orientation and Goal Prioritization: This stage concerns the emergence of an approach motivation toward pro-social action. The evidence for this component is more tentative; however, it is partially supported by a small but consistent body of EEG studies showing greater left frontal alpha asymmetry (e.g., Huffmeijer et al., 2012) and fMRI findings implicating the vmPFC/dlPFC in goal-directed choice (e.g., Weng et al., 2013).
4. Cognitive Control and Decision Resolution: When a conflict arises between selfish and altruistic impulses, cognitive control is engaged. This is strongly supported by extensive fMRI and EEG findings showing recruitment of the dlPFC and ACC, along with increased Fmθ during conflict-laden decisions (e.g., Hu et al., 2021; Rodrigues et al., 2015; Weng et al., 2013).
5. Behavioral Implementation and Outcome Monitoring: The final component involves executing the action and evaluating its outcome. This is supported by EEG-ERP studies demonstrating modulation of feedback-related components like the P300 and FRN in response to altruistic outcomes (e.g., Gan et al., 2022; Luo et al., 2019).

To reiterate, this framework is a conceptual heuristic designed to organize diverse findings and stimulate testable hypotheses. The novelty of this framework lies in its attempt to explicitly map reported neurophysiological correlates from multiple modalities onto these conceptual components of altruistic motivation, while fully acknowledging its speculative nature and the substantial research needed for validation. The primary aim here is to provide an organizational structure for the diverse neurophysiological findings related to costly, other-oriented altruistic motivation, rather than to assert a confirmed, step-by-step causal model. The “novelty” of this framework lies in its attempt to explicitly map reported neurophysiological correlates from multiple modalities onto these conceptual components of altruistic motivation, while fully acknowledging its speculative nature and the substantial research needed for validation.

### Clinical and societal implications

While this review focused on the neurophysiology of altruism primarily in healthy populations, a deeper understanding of these mechanisms could, in the very long term and after substantial further research, inform avenues for investigating social cognitive functioning in various conditions. Future research might explore whether understanding the typical functioning of these circuits in altruism could eventually contribute to a more nuanced understanding of social processing differences in conditions such as autism spectrum disorder or schizophrenia. Similarly, the observation that altruistic actions can engage reward circuitry might, again with extensive future research, offer novel exploratory perspectives for investigating anhedonia or motivational deficits, for instance, in depression. Non-invasive brain stimulation techniques (e.g., tDCS, TMS) targeting specific neural oscillatory patterns or connectivity are being explored in basic research to modulate social cognitive processes, but their potential to therapeutically modulate altruistic tendencies is highly speculative and requires extensive investigation.

It is crucial to emphasize that any potential translation to clinical understanding or application is extremely premature and speculative at this stage. The findings of this scoping review are descriptive, do not establish causal links between the identified neural patterns and behavior, and certainly do not provide evidence that these patterns are causally related to therapeutic outcomes or that interventions based on them would be effective or safe. Nonetheless, some additional considerations warrant explicit mention to further clarify the extent of current limitations in translating social neuroscience findings to clinical practice. First, the issue of specificity remains a major challenge: many neurophysiological markers of altruistic motivation, such as activity within the medial prefrontal cortex or the temporoparietal junction, are not unique to altruism but are instead implicated across a range of social cognitive and affective processes. This overlap constrains their diagnostic value and limits their clinical applicability. Second, reported effect sizes associated with neural and physiological signatures of altruistic behavior are typically modest, often reflecting group-level tendencies rather than robust indicators at the individual level. As such, their utility for prediction, stratification, or intervention planning in clinical settings remains extremely limited. Finally, questions of ecological validity persist, as the vast majority of empirical studies reviewed were conducted in highly controlled experimental environments employing abstract and decontextualized paradigms. These conditions may not accurately reflect the complexity, heterogeneity, and contextual embedding of altruistic behavior in real-world social and clinical contexts. Together, these limitations underscore that while current research maps important directions for future translational science, meaningful clinical impact will require not only stronger evidence for causality and effect size but also greater specificity and ecological robustness in both measurement and interpretation.

#### Strengths and limitations

This scoping review possesses several strengths, including its focused search strategy across multiple databases, adherence to PRISMA-ScR guidelines, and the systematic mapping of a diverse range of neurophysiological studies on altruism. By synthesizing findings across fMRI, EEG, fNIRS, and ANS modalities, it provides a multifaceted overview of the current research landscape and explicitly identifies the relative contributions and limitations of each within this specific topic. In particular, we note that conclusions regarding fNIRS are necessarily tentative given the extremely limited evidence base (single study) presently available. While PRISMA-ScR does not require formal risk-of-bias assessment, we note that most included neuroimaging studies had modest sample sizes, variable task designs, and diverse analytic strategies. These limitations reduce the generalizability of patterns and detail the infeasibility, at present, of robust meta-analytic synthesis. Furthermore, the lack of standardized reporting and the predominance of samples drawn from WEIRD (Western, Educated, Industrialized, Rich, and Democratic) populations constrain the global external validity of the synthesized patterns. These challenges render formal meta-analytic aggregation and quantitative comparison currently infeasible. We emphasize, therefore, that the findings presented here should be interpreted as an initial qualitative synthesis reflective of present reporting practices, and that refinement and further generalization will require future, methodologically harmonized, and demographically diverse studies designed to enable robust meta-analytic integration.

Additionally, several limitations must be acknowledged. First, the predominance of laboratory-based paradigms (e.g., Dictator Game, donation tasks), while offering experimental control, may limit the ecological validity of some findings concerning real-world altruistic behavior. Second, the operationalization of altruism and its associated costs varied considerably across studies, making direct comparisons challenging and necessitating caution in synthesizing results. A key limitation of the current literature, and thus this review, is the significant heterogeneity in the operationalization of altruistic “cost” and experimental paradigms. While we systematically catalogued these differences in Table 1 to guide interpretation, this variability constrains direct comparisons and currently precludes formal meta-analytic synthesis. Therefore, our conclusions should be seen as a qualitative mapping of a diverse field, with claims about unified neural mechanisms remaining provisional until greater methodological standardization is achieved. Further, although we took rigorous measures to classify paradigms as “core altruistic” or “borderline/strategic” according to predefined criteria, it should be noted that the operational boundaries between these categories can be ambiguous in certain cases. Some paradigms lack clearly specified self-costs or identifiable other-benefits, resulting in their conservative classification as “borderline/strategic.” The neurophysiological findings from such paradigms may not directly reflect altruistic motivation per se, but rather broader prosociality or mixed-motive behavior. This limitation underscores the need for cautious interpretation of these results and motivates future work towards finer conceptual and methodological granularity in paradigm design and classification. The heterogeneity of altruism paradigms – encompassing both empathy-driven and fairness-based tasks – is documented, though not systematically dissected in the present synthesis. Third, as highlighted in our Results, the number of studies focusing primarily on ANS activity was relatively small, restricting the robustness of conclusions regarding specific autonomic signatures of altruism. Fourth, the synthesis is predominantly based on studies with healthy adult populations. While the inclusion criteria were broad, the final set of studies did not yield a substantial body of research on the impact of specific clinical disorders on the neurobiology of altruism, thus limiting generalizability to such populations. Fifth, the literature search for this review concluded on 31 December 2024. Consequently, studies published after this date are not included. Finally, as a scoping review, this work did not involve a formal assessment of the methodological quality or risk of bias of individual studies, nor did it undertake quantitative meta-analysis or effect size calculations. The summarized findings, therefore, represent a qualitative mapping and narrative synthesis of the available evidence.

The lack of tDCS, neurofeedback, and trait-level studies is not due to our design but the outcome of our specific search terms and selection criteria. Future reviews using different definitions/interfaces may yield different results.

#### Future directions

Building upon the insights and limitations identified in this review, several avenues for future research are proposed:

- Enhancing ecological validity: We hypothesize that immersive paradigms (e.g., virtual reality) will reveal stronger engagement of the amygdala and insula during altruistic acts compared to conventional laboratory tasks, reflecting greater emotional salience.
- Investigating developmental trajectories: Longitudinal studies can test the hypothesis that the neural basis of altruism shifts from primarily reward-based subcortical activation in childhood to greater recruitment of PFC and mentalizing (TPJ) networks in adolescence, as cognitive control and perspective-taking mature.
- Cross-cultural neuroscientific research: We hypothesize that individuals from collectivist cultures will display greater mPFC-TPJ functional connectivity when making altruistic decisions benefitting in-group members, compared to those from individualist cultures – reflecting heightened social cognitive integration driven by cultural values. Future studies can directly test this by combining cultural priming with neuroimaging.
- Advancing autonomic research: Multi-modal studies should test the hypothesis that a state of high parasympathetic tone (indexed by HRV) precedes and correlates with increased left-frontal EEG asymmetry during altruistic choices, suggesting that physiological calm facilitates approach-oriented prosocial motivation.
- Exploring individual differences: Research should test the hypothesis that distinct neural pathways support altruism based on personality. For instance, we predict that trait empathy will correlate with greater anterior insula activation, whereas trait conscientiousness will correlate with stronger dlPFC engagement during costly helping.
- Causal investigations and interventions: Non-invasive brain stimulation allows for causal tests. We predict that applying inhibitory TMS to the right TPJ will selectively reduce the amount of money given in a Dictator Game, without altering basic reward processing, thereby confirming the region’s causal role in altruistic valuation.
- Integrating computational modeling: Future work should test the hypothesis that developmental and cultural factors parametrically modulate the relative weighting of self- versus other-regarding value signals in the vmPFC. This can be specified by fitting hierarchical Bayesian models to neural and behavioral data from diverse populations.

The very limited fNIRS evidence (single study) highlights a potential emerging avenue: fNIRS may bridge the gap between the spatial resolution of fMRI and the temporal resolution of EEG, particularly in ecologically valid, real-world contexts. We encourage further multimodal and cross-cultural work to clarify and unify findings across different paradigms, populations, and neurophysiological techniques.

## Conclusion

This scoping review has meticulously synthesized the current neurophysiological understanding of altruistic motivation, illuminating a remarkably complex and multifaceted interplay of cognitive, affective, and decision-making processes. These processes are subserved by a distributed, dynamically interacting network of brain regions and are suggested by the reviewed literature to be potentially coupled with peripheral physiological responses. While fMRI has provided a foundational spatial map, and EEG and ANS research are increasingly revealing the crucial temporal and embodied dimensions, the field is vibrant and poised for transformative discoveries. Furthering a more profound, nuanced, and integrative understanding of the neurobiological mechanisms that enable and motivate human altruism enriches our fundamental scientific comprehension of one of our species’ most cherished capacities. The synthesized findings may, in the long term, offer foundational knowledge relevant to future investigations aimed at understanding factors that could contribute to individual well-being and social cohesion. Achieving such societal benefits, however, will necessitate extensive further research, including the development and rigorous testing of any potential applications, alongside careful consideration of ethical implications. The continued pursuit of this endeavor, demanding interdisciplinary collaboration, methodological innovation, critical appraisal of evidence, and unwavering ethical vigilance, will be paramount in advancing this vital frontier of human neuroscience.

## Supplementary Information

Below is the link to the electronic supplementary material.

Supplementary file1 (DOCX 35 KB)

## Data Availability

Data sharing is not applicable to this article as no datasets were generated or analyzed during the current study. All data sources are from previously published studies, which are cited in the reference list.

## Declarations

### Open practices statement

A registered protocol is not required for scoping review

## References

[CR1] Bandura, A. (1986). *Social foundations of thought and action: A social cognitive theory* (pp. xiii–617). Prentice-Hall, Inc.

[CR2] Bas, L. M., Roberts, I. D., Hutcherson, C. A., & Tusche, A. (2023). A neurocomputational account of the link between social perception and social action. *eLife,**12*, Article RP92539. 10.7554/eLife.92539.1 10.7554/eLife.92539 PMC12002797 40237179

[CR3] Batson, C. D. (2011). *Altruism in humans* (pp. vi–329). Oxford University Press.

[CR4] Batson, C. D., Duncan, B. D., Ackerman, P., Buckley, T., & Birch, K. (1981). Is empathic emotion a source of altruistic motivation? *Journal of Personality and Social Psychology,**40*(2), 290–302. 10.1037/0022-3514.40.2.290

[CR5] Bellucci, G., Camilleri, J. A., Eickhoff, S. B., & Krueger, F. (2020). Neural signatures of prosocial behaviors. *Neuroscience and Biobehavioral Reviews,**118*, 186–195. 10.1016/j.neubiorev.2020.07.006 32707344 10.1016/j.neubiorev.2020.07.006 PMC7958651

[CR6] Boccadoro, S., Wagels, L., Puiu, A. A., Votinov, M., Weidler, C., Veselinovic, T., Demko, Z., Raine, A., & Neuner, I. (2021). A meta-analysis on shared and distinct neural correlates of the decision-making underlying altruistic and retaliatory punishment. *Human Brain Mapping,**42*(17), 5547–5562. 10.1002/hbm.25635 34415078 10.1002/hbm.25635 PMC8559514

[CR7] Böckler, A., Tusche, A., Schmidt, P., & Singer, T. (2018). Distinct mental trainings differentially affect altruistically motivated, norm motivated, and self-reported prosocial behaviour. *Scientific Reports,**8*(1), 13560. 10.1038/s41598-018-31813-8 30202029 10.1038/s41598-018-31813-8 PMC6131389

[CR8] Camerer, C. F. (2010). Behavioural game theory. In S. N. Durlauf & L. E. Blume (Eds.), *Behavioural and Experimental Economics* (pp. 42–50). Palgrave Macmillan UK. 10.1057/9780230280786_6

[CR9] Caspers, S., Heim, S., Lucas, M. G., Stephan, E., Fischer, L., Amunts, K., & Zilles, K. (2011). Moral concepts set decision strategies to abstract values. *PLoS ONE,**6*(4), Article e18451. 10.1371/journal.pone.0018451 21483767 10.1371/journal.pone.0018451 PMC3069966

[CR10] Cavanagh, J. F., Eisenberg, I., Guitart-Masip, M., Huys, Q., & Frank, M. J. (2013). Frontal theta overrides Pavlovian learning biases. *The Journal of Neuroscience,**33*(19), 8541–8548. 10.1523/JNEUROSCI.5754-12.2013 23658191 10.1523/JNEUROSCI.5754-12.2013 PMC3707146

[CR11] Clavien, C., & Chapuisat, M. (2013). Altruism across disciplines: One word, multiple meanings. *Biology & Philosophy,**28*(1), 125–140. 10.1007/s10539-012-9317-3

[CR12] Coan, J. A., & Allen, J. J. B. (2003). Frontal EEG asymmetry and the behavioral activation and inhibition systems. *Psychophysiology,**40*(1), 106–114. 10.1111/1469-8986.00011 12751808 10.1111/1469-8986.00011

[CR13] Correa, K. A., Stone, B. T., Stikic, M., Johnson, R. R., & Berka, C. (2015). Characterizing donation behavior from psychophysiological indices of narrative experience. *Frontiers in Neuroscience,**9*, Article 301. 10.3389/fnins.2015.00301 26379488 10.3389/fnins.2015.00301 PMC4553387

[CR14] Cutler, J., & Campbell-Meiklejohn, D. (2019). A comparative fMRI meta-analysis of altruistic and strategic decisions to give. *NeuroImage,**184*, 227–241. 10.1016/j.neuroimage.2018.09.009 30195947 10.1016/j.neuroimage.2018.09.009

[CR15] Deci, E. L., & Ryan, R. M. (1985). *Intrinsic motivation and self-determination in human behavior*. Springer. 10.1007/978-1-4899-2271-7

[CR16] Eisenberg, N., Fabes, R. A., Miller, P. A., Fultz, J., Shell, R., Mathy, R. M., & Reno, R. R. (1989). Relation of sympathy and personal distress to prosocial behavior: A multimethod study. *Journal of Personality and Social Psychology,**57*(1), 55–66. 10.1037/0022-3514.57.1.55 2754604 10.1037//0022-3514.57.1.55

[CR17] Eisenberg, N., & Miller, P. A. (1987). The relation of empathy to prosocial and related behaviors. *Psychological Bulletin,**101*(1), 91–119. 3562705

[CR18] Fede, S. J., Pearson, E. E., Kerich, M., & Momenan, R. (2021). Charity preferences and perceived impact moderate charitable giving and associated neural response. *Neuropsychologia,**160*, Article 107957. 10.1016/j.neuropsychologia.2021.107957 34271001 10.1016/j.neuropsychologia.2021.107957 PMC11044562

[CR19] Fehr, E., & Fischbacher, U. (2003). The nature of human altruism. *Nature,**425*(6960), 785–791. 10.1038/nature02043 14574401 10.1038/nature02043

[CR20] Fetchenhauer, D., & Bierhoff, H.-W. (2004). Altruismus aus evolutionstheoretischer Perspektive. *Zeitschrift für Sozialpsychologie,**35*(3), 131–141. 10.1024/0044-3514.35.3.131

[CR21] Filkowski, M. M., Cochran, R. N., & Haas, B. W. (2016). Altruistic behavior: Mapping responses in the brain. *Neuroscience and Neuroeconomics,**5*, 65–75. 10.2147/NAN.S87718 28580317 10.2147/NAN.S87718 PMC5456281

[CR22] Gan, T., Zhang, Y., Zhang, L., & Gu, R. (2022). Neural sensitivity to helping outcome predicts helping decision in real life. *Neuropsychologia,**173*, Article 108291. 10.1016/j.neuropsychologia.2022.108291 35690115 10.1016/j.neuropsychologia.2022.108291

[CR23] Goetz, J. L., Keltner, D., & Simon-Thomas, E. (2010). Compassion: An evolutionary analysis and empirical review. *Psychological Bulletin,**136*(3), 351–374. 10.1037/a0018807 20438142 10.1037/a0018807 PMC2864937

[CR24] Greening, S., Norton, L., Virani, K., Ty, A., Mitchell, D., & Finger, E. (2014). Individual differences in the anterior insula are associated with the likelihood of financially helping versus harming others. *Cognitive, Affective, & Behavioral Neuroscience,**14*(1), 266–277. 10.3758/s13415-013-0213-3 10.3758/s13415-013-0213-3 24097059

[CR25] Haas, B. W., Brook, M., Remillard, L., Ishak, A., Anderson, I. W., & Filkowski, M. M. (2015). I know how you feel: The warm-altruistic personality profile and the empathic brain. *PLoS ONE,**10*(3), Article e0120639. 10.1371/journal.pone.0120639 25769028 10.1371/journal.pone.0120639 PMC4359130

[CR26] Haber, S. N., & Knutson, B. (2010). The reward circuit: Linking primate anatomy and human imaging. *Neuropsychopharmacology,**35*(1), 4–26. 10.1038/npp.2009.129 19812543 10.1038/npp.2009.129 PMC3055449

[CR27] Harbaugh, W. T., Mayr, U., & Burghart, D. R. (2007). Neural responses to taxation and voluntary giving reveal motives for charitable donations. *Science,**316*(5831), 1622–1625. 10.1126/science.1140738 17569866 10.1126/science.1140738

[CR28] Hare, T. A., Camerer, C. F., Knoepfle, D. T., O’Doherty, J. P., & Rangel, A. (2010). Value computations in ventral medial prefrontal cortex during charitable decision making incorporate input from regions involved in social cognition. *The Journal of Neuroscience,**30*(2), 583–590. 10.1523/JNEUROSCI.4089-09.2010 20071521 10.1523/JNEUROSCI.4089-09.2010 PMC6633003

[CR29] Harmon-Jones, E., & Allen, J. J. B. (1997). Behavioral activation sensitivity and resting frontal EEG asymmetry: Covariation of putative indicators related to risk for mood disorders. *Journal of Abnormal Psychology,**106*(1), 159–163. 10.1037/0021-843X.106.1.159 9103728 10.1037//0021-843x.106.1.159

[CR30] Himichi, T., & Nomura, M. (2015). Modulation of empathy in the left ventrolateral prefrontal cortex facilitates altruistic behavior: An fNIRS study. *Journal of Integrative Neuroscience,**14*(02), 207–222. 10.1142/S0219635215500120 25903500 10.1142/S0219635215500120

[CR31] Hu, J., Hu, Y., Li, Y., & Zhou, X. (2021). Computational and neurobiological substrates of cost-benefit integration in altruistic helping decision. *Journal of Neuroscience,**41*(15), 3545–3561. 10.1523/JNEUROSCI.1939-20.2021 33674417 10.1523/JNEUROSCI.1939-20.2021 PMC8051690

[CR32] Hu, J., Konovalov, A., & Ruff, C. C. (2023). A unified neural account of contextual and individual differences in altruism. *eLife,**12*, Article e80667. 10.7554/eLife.80667 36752704 10.7554/eLife.80667 PMC9908080

[CR33] Hu, Y., Strang, S., & Weber, B. (2015). Helping or punishing strangers: Neural correlates of altruistic decisions as third-party and of its relation to empathic concern. *Frontiers in Behavioral Neuroscience*, *9*. 10.3389/fnbeh.2015.00024 10.3389/fnbeh.2015.00024 PMC4332347 25741254

[CR34] Huang, Q., Li, D., Zhou, C., Xu, Q., Li, P., & Warren, C. M. (2021). Multivariate pattern analysis of electroencephalography data reveals information predictive of charitable giving. *NeuroImage,**242*, Article 118475. 10.1016/j.neuroimage.2021.118475 34403743 10.1016/j.neuroimage.2021.118475

[CR35] Hubbard, J., Harbaugh, W. T., Srivastava, S., Degras, D., & Mayr, U. (2016). A general benevolence dimension that links neural, psychological, economic, and life-span data on altruistic tendencies. *Journal of Experimental Psychology: General,**145*(10), 1351–1358. 10.1037/xge0000209 27513302 10.1037/xge0000209 PMC5585009

[CR36] Huffmeijer, R., Alink, L. R. A., Tops, M., Bakermans-Kranenburg, M. J., & van IJzendoorn, M. H. (2012). Asymmetric frontal brain activity and parental rejection predict altruistic behavior: Moderation of oxytocin effects. *Cognitive, Affective & Behavioral Neuroscience,**12*(2), 382–392. 10.3758/s13415-011-0082-6 10.3758/s13415-011-0082-6 PMC3341522 22246695

[CR37] Hutcherson, C. A., Bushong, B., & Rangel, A. (2015). A neurocomputational model of altruistic choice and its implications. *Neuron,**87*(2), 451–462. 10.1016/j.neuron.2015.06.031 26182424 10.1016/j.neuron.2015.06.031 PMC4947370

[CR38] Ishii, R., Shinosaki, K., Ukai, S., Inouye, T., Ishihara, T., Yoshimine, T., Hirabuki, N., Asada, H., Kihara, T., Robinson, S. E., & Takeda, M. (1999). Medial prefrontal cortex generates frontal midline theta rhythm. *Neuroreport,**10*(4), 675–679. 10.1097/00001756-199903170-00003 10208529 10.1097/00001756-199903170-00003

[CR39] Karns, C. M., Moore, W. E., & Mayr, U. (2017). The cultivation of pure altruism via gratitude: A functional MRI study of change with gratitude practice. *Frontiers in Human Neuroscience,**11*, 599. 10.3389/fnhum.2017.00599 29375336 10.3389/fnhum.2017.00599 PMC5770643

[CR40] Kirk, U., Gu, X., Sharp, C., Hula, A., Fonagy, P., & Montague, P. R. (2016). Mindfulness training increases cooperative decision making in economic exchanges: Evidence from fMRI. *NeuroImage,**138*, 274–283. 10.1016/j.neuroimage.2016.05.075 27266443 10.1016/j.neuroimage.2016.05.075 PMC4954868

[CR41] Klimecki, O. M., Leiberg, S., Ricard, M., & Singer, T. (2014). Differential pattern of functional brain plasticity after compassion and empathy training. *Social Cognitive and Affective Neuroscience,**9*(6), 873–879. 10.1093/scan/nst060 23576808 10.1093/scan/nst060 PMC4040103

[CR42] Kuss, K., Falk, A., Trautner, P., Elger, C. E., Weber, B., & Fliessbach, K. (2013). A reward prediction error for charitable donations reveals outcome orientation of donators. *Social Cognitive and Affective Neuroscience,**8*(2), 216–223. 10.1093/scan/nsr088 22198972 10.1093/scan/nsr088 PMC3575724

[CR43] Kwon, S.-J., Van Hoorn, J., Do, K. T., Burroughs, M., & Telzer, E. H. (2023). Neural representation of donating time and money. *Journal of Neuroscience,**43*(36), 6297–6305. 10.1523/JNEUROSCI.0480-23.2023 37580120 10.1523/JNEUROSCI.0480-23.2023 PMC10490462

[CR44] Lay, J. C., & Hoppmann, C. A. (2015). Altruism and Prosocial Behavior. In N. A. Pachana (Ed.), *Encyclopedia of Geropsychology* (pp. 1–9). Springer Singapore. 10.1007/978-981-287-080-3_69-1

[CR45] Lazić, A., Purić, D., & Krstić, K. (2021). Does parochial cooperation exist in childhood and adolescence? A meta-analysis. *International Journal of Psychology,**56*(6), 917–933. 10.1002/ijop.12791 34212370 10.1002/ijop.12791

[CR46] Liu, X., Hu, X., Shi, K., & Mai, X. (2020). Your losses are mine: The influence of empathic concern on evaluative processing of others’ outcomes. *Cognitive, Affective & Behavioral Neuroscience,**20*(3), 481–492. 10.3758/s13415-020-00779-4 10.3758/s13415-020-00779-4 32124255

[CR47] Lockwood, P. L., Apps, M. A. J., Valton, V., Viding, E., & Roiser, J. P. (2016). Neurocomputational mechanisms of prosocial learning and links to empathy. *Proceedings of the National Academy of Sciences of the United States of America,**113*(35), 9763–9768. 10.1073/pnas.1603198113 27528669 10.1073/pnas.1603198113 PMC5024617

[CR48] Luo, Y., Jiang, H., Chen, X., Zhang, Y., & You, X. (2019). Temporal dynamics of hedonic and eudaimonic reward processing: An event-related potentials (ERPs) study. *International Journal of Psychophysiology,**137*, 63–71. 10.1016/j.ijpsycho.2018.12.009 30576767 10.1016/j.ijpsycho.2018.12.009

[CR49] Luo, Y., Zhang, X., Jiang, H., & Chen, X. (2022). The neural habituation to hedonic and eudaimonic rewards: Evidence from reward positivity. *Psychophysiology,**59*(3), Article e13977. 10.1111/psyp.13977 34846754 10.1111/psyp.13977

[CR50] Mao, W., Xiao, Q., Shen, X., Zhou, X., Wang, A., & Jin, J. (2024). How effort-based self-interest motivation shapes altruistic donation behavior and brain responses. *Psychophysiology,**61*(7), Article e14552. 10.1111/psyp.14552 38406999 10.1111/psyp.14552

[CR51] Mitiureva, D. G., Terlichenko, E. O., Zubko, V. M., Kabanova, P. I., Abrosimova, V. D., Skripkina, S. M., Krivchenkova, E. V., Verkholaz, D. M., Borodkina, A. S., Komarova, A. V., & Kiselnikov, A. A. (2024). Neural mechanisms of altruistic decision-making: EEG functional connectivity network analysis. *Cognitive, Affective & Behavioral Neuroscience,**24*(6), 1109–1120. 10.3758/s13415-024-01214-8 10.3758/s13415-024-01214-8 39198301

[CR52] Moll, J., Krueger, F., Zahn, R., Pardini, M., De Oliveira-Souza, R., & Grafman, J. (2006). Human fronto–mesolimbic networks guide decisions about charitable donation. *Proceedings of the National Academy of Sciences of the United States of America,**103*(42), 15623–15628. 10.1073/pnas.0604475103 17030808 10.1073/pnas.0604475103 PMC1622872

[CR53] Narveson, J. (2012). Egoism and Altruism. In *Encyclopedia of Applied Ethics* (pp. 51–55). Elsevier. 10.1016/B978-0-12-373932-2.00199-X

[CR54] Orui, J., Shiraiwa, K., Inoue, T., Ueda, M., Ueno, K., Naito, Y., & Ishii, R. (2025). Neurophysiological effects of altruistically motivated craft activities in occupational therapy: A pilot study using frontal EEG and heart rate variability analysis. *Hong Kong Journal of Occupational Therapy*. 10.1177/15691861251319466 39975471 10.1177/15691861251319466 PMC11833797

[CR55] Oyediran, O. A., & Rivas, M. F. (2017). Response time and heart rate in a moral dilemma. *Journal of Neuroscience, Psychology, and Economics,**10*(1), 42–58. 10.1037/npe0000071

[CR56] Pfattheicher, S., Nielsen, Y. A., & Thielmann, I. (2022). Prosocial behavior and altruism: A review of concepts and definitions. *Current Opinion in Psychology,**44*, 124–129. 10.1016/j.copsyc.2021.08.021 34627110 10.1016/j.copsyc.2021.08.021

[CR57] Phan, K. L., Wager, T., Taylor, S. F., & Liberzon, I. (2002). Functional neuroanatomy of emotion: A meta-analysis of emotion activation studies in PET and fMRI. *NeuroImage,**16*(2), 331–348. 10.1006/nimg.2002.1087 12030820 10.1006/nimg.2002.1087

[CR58] Piva, M., Velnoskey, K., Jia, R., Nair, A., Levy, I., & Chang, S. W. (2019). The dorsomedial prefrontal cortex computes task-invariant relative subjective value for self and other. *eLife,**8*, Article e44939. 10.7554/eLife.44939 31192786 10.7554/eLife.44939 PMC6565363

[CR59] Porges, S. W. (2011). *The polyvagal theory: Neurophysiological foundations of emotions, attachment, communication, and self-regulation* (1st ed). Norton.

[CR60] Post, S. G. (2005). Altruism, happiness, and health: It’s good to be good. *International Journal of Behavioral Medicine,**12*(2), 66–77. 10.1207/s15327558ijbm1202_4 15901215 10.1207/s15327558ijbm1202_4

[CR61] Rahimyar, A., & Sarvari, H. (2023). The impacts of altruism on the individual and society. *Journal of Humanities and Social Sciences Studies*. 10.32996/jhsss.2023.5.4.4

[CR62] Rhoads, S. A., O’Connell, K., Berluti, K., Ploe, M. L., Elizabeth, H. S., Amormino, P., Li, J. L., Dutton, M. A., VanMeter, A. S., & Marsh, A. A. (2023). Neural responses underlying extraordinary altruists’ generosity for socially distant others. *PNAS Nexus,**2*(7), Article pgad199. 10.1093/pnasnexus/pgad199 37416875 10.1093/pnasnexus/pgad199 PMC10321390

[CR63] Rilling, J. K., DeMarco, A. C., Hackett, P. D., Chen, X., Gautam, P., Stair, S., Haroon, E., Thompson, R., Ditzen, B., Patel, R., & Pagnoni, G. (2014). Sex differences in the neural and behavioral response to intranasal oxytocin and vasopressin during human social interaction. *Psychoneuroendocrinology,**39*, 237–248. 10.1016/j.psyneuen.2013.09.022 24157401 10.1016/j.psyneuen.2013.09.022 PMC3842401

[CR64] Rilling, J. K., Gutman, D. A., Zeh, T. R., Pagnoni, G., Berns, G. S., & Kilts, C. D. (2002). A neural basis for social cooperation. *Neuron,**35*(2), 395–405. 10.1016/S0896-6273(02)00755-9 12160756 10.1016/s0896-6273(02)00755-9

[CR65] Rodrigues, J., Ulrich, N., & Hewig, J. (2015). A neural signature of fairness in altruism: A game of theta? *Social Neuroscience,**10*(2), 192–205. 10.1080/17470919.2014.977401 25350461 10.1080/17470919.2014.977401

[CR66] Ruff, C. C., & Fehr, E. (2014). The neurobiology of rewards and values in social decision making. *Nature Reviews Neuroscience,**15*(8), 549–562. 10.1038/nrn3776 24986556 10.1038/nrn3776

[CR67] San Martín, R., Kwak, Y., Pearson, J. M., Woldorff, M. G., & Huettel, S. A. (2016). Altruistic traits are predicted by neural responses to monetary outcomes for self vs charity. *Social Cognitive and Affective Neuroscience,**11*(6), 863–876. 10.1093/scan/nsw026 27030510 10.1093/scan/nsw026 PMC4884320

[CR68] Schroeder, D. A., Graziano, W. G., Batson, C. D., Lishner, D. A., & Stocks, E. L. (2015). The Empathy–Altruism Hypothesis. In D. A. Schroeder & W. G. Graziano (Eds.), *The Oxford Handbook of Prosocial Behavior*. Oxford University Press. 10.1093/oxfordhb/9780195399813.013.023

[CR69] Schulreich, S., Tusche, A., Kanske, P., & Schwabe, L. (2022). Altruism under stress: Cortisol negatively predicts charitable giving and neural value representations depending on mentalizing capacity. *The Journal of Neuroscience,**42*(16), 3445–3460. 10.1523/JNEUROSCI.1870-21.2022 35288436 10.1523/JNEUROSCI.1870-21.2022 PMC9034777

[CR70] Schulreich, S., Tusche, A., Kanske, P., & Schwabe, L. (2023). Higher subjective socioeconomic status is linked to increased charitable giving and mentalizing-related neural value coding. *NeuroImage,**279*, Article 120315. 10.1016/j.neuroimage.2023.120315 37557972 10.1016/j.neuroimage.2023.120315

[CR71] Sellitto, M., Neufang, S., Schweda, A., Weber, B., & Kalenscher, T. (2021). Arbitration between insula and temporoparietal junction subserves framing-induced boosts in generosity during social discounting. *NeuroImage,**238*, Article 118211. 10.1016/j.neuroimage.2021.118211 34116152 10.1016/j.neuroimage.2021.118211

[CR72] Sober, E., & Wilson, D. S. (1998). *Unto others: The evolution and psychology of unselfish behavior* (p. 394). Harvard University Press.

[CR73] Stellar, J. E., Cohen, A., Oveis, C., & Keltner, D. (2015). Affective and physiological responses to the suffering of others: Compassion and vagal activity. *Journal of Personality and Social Psychology,**108*(4), 572–585. 10.1037/pspi0000010 25621856 10.1037/pspi0000010

[CR74] Stocks, E. L., & Lishner, D. A. (2023). Altruism. In H. S. Friedman & C. H. Markey (Eds.), *Encyclopedia of Mental Health (Third Edition)* (pp. 62–67). Academic Press. 10.1016/B978-0-323-91497-0.00105-3

[CR75] Sul, S., Tobler, P. N., Hein, G., Leiberg, S., Jung, D., Fehr, E., & Kim, H. (2015). Spatial gradient in value representation along the medial prefrontal cortex reflects individual differences in prosociality. *Proceedings of the National Academy of Sciences of the United States of America,**112*(25), 7851–7856. 10.1073/pnas.1423895112 26056280 10.1073/pnas.1423895112 PMC4485092

[CR76] Tamir, D. I., Zaki, J., & Mitchell, J. P. (2015). Informing others is associated with behavioral and neural signatures of value. *Journal of Experimental Psychology: General,**144*(6), 1114–1123. 10.1037/xge0000122 26595840 10.1037/xge0000122

[CR77] Tousignant, B., Eugène, F., Sirois, K., & Jackson, P. L. (2018). Difference in neural response to social exclusion observation and subsequent altruism between adolescents and adults. *Neuropsychologia,**116*, 15–25. 10.1016/j.neuropsychologia.2017.04.017 28412511 10.1016/j.neuropsychologia.2017.04.017

[CR78] Tricco, A. C., Lillie, E., Zarin, W., O’Brien, K. K., Colquhoun, H., Levac, D., Moher, D., Peters, M. D. J., Horsley, T., Weeks, L., Hempel, S., Akl, E. A., Chang, C., McGowan, J., Stewart, L., Hartling, L., Aldcroft, A., Wilson, M. G., Garritty, C., … Straus, S. E. (2018). PRISMA Extension for Scoping Reviews (PRISMA-ScR): Checklist and Explanation. *Annals of Internal Medicine*, *169*(7), 467–473. 10.7326/M18-0850 10.7326/M18-0850 30178033

[CR79] Tusche, A., Böckler, A., Kanske, P., Trautwein, F.-M., & Singer, T. (2016). Decoding the charitable brain: Empathy, perspective taking, and attention shifts differentially predict altruistic giving. *The Journal of Neuroscience,**36*(17), 4719–4732. 10.1523/JNEUROSCI.3392-15.2016 27122031 10.1523/JNEUROSCI.3392-15.2016 PMC6601722

[CR80] Van Overwalle, F. (2009). Social cognition and the brain: A meta-analysis. *Human Brain Mapping,**30*(3), 829–858. 10.1002/hbm.20547 18381770 10.1002/hbm.20547 PMC6870808

[CR81] Weng, H. Y., Fox, A. S., Shackman, A. J., Stodola, D. E., Caldwell, J. Z. K., Olson, M. C., Rogers, G. M., & Davidson, R. J. (2013). Compassion training alters altruism and neural responses to suffering. *Psychological Science,**24*(7), 1171–1180. 10.1177/0956797612469537 23696200 10.1177/0956797612469537 PMC3713090

[CR82] West, S. A., Griffin, A. S., & Gardner, A. (2007). Evolutionary explanations for cooperation. *Current Biology,**17*(16), R661–R672. 10.1016/j.cub.2007.06.004 17714660 10.1016/j.cub.2007.06.004

[CR83] Wijaya, V. G., Oba, K., Ishibashi, R., & Sugiura, M. (2023). Why people hesitate to help: Neural correlates of the counter-dynamics of altruistic helping and individual differences in daily helping tendencies. *Frontiers in Psychology,**14*, 1080376. 10.3389/fpsyg.2023.1080376 36998358 10.3389/fpsyg.2023.1080376 PMC10044345

[CR84] Wu, Y. E., & Hong, W. (2022). Neural basis of prosocial behavior. *Trends in Neurosciences,**45*(10), 749–762. 10.1016/j.tins.2022.06.008 35853793 10.1016/j.tins.2022.06.008 PMC10039809

[CR85] Zaki, J., López, G., & Mitchell, J. P. (2014). Activity in ventromedial prefrontal cortex co-varies with revealed social preferences: Evidence for person-invariant value. *Social Cognitive and Affective Neuroscience,**9*(4), 464–469. 10.1093/scan/nst005 23314009 10.1093/scan/nst005 PMC3989125

[CR86] Zanon, M., Novembre, G., Zangrando, N., Chittaro, L., & Silani, G. (2014). Brain activity and prosocial behavior in a simulated life-threatening situation. *NeuroImage,**98*, 134–146. 10.1016/j.neuroimage.2014.04.053 24780697 10.1016/j.neuroimage.2014.04.053

