# Supplementary material for: The neurophysiological correlates of altruism: A scoping review of fMRI, EEG, and autonomic studies
Source: Cogn Affect Behav Neurosci. 2025 Dec 17;26(4):1452–77. doi: 10.3758/s13415-025-01380-3 (PMC13385233; doi:10.3758/s13415-025-01380-3)
Supplement: Supplementary file 1 — Supplementary file1 (DOCX 35 KB) [file 13415_2025_1380_MOESM1_ESM.docx]

| Article | Reason for Exclusion |
| --- | --- |
| Rilling et al., 2008 | Not strictly altruism, but cooperation focus |
| Mathur et al., 2010 | Only observation, not action |
| Gospic et al., 2014 | Focus is on validation, not altruism itself |
| FeldmanHall et al., 2015 | Focus is on empathy, not direct altruistic acts |
| Telzer et al., 2015 | Not core altruism, group bias focus |
| Brunnlieb et al., 2016 | Cooperation, not altruism |
| David et al., 2017 | Focus is on comparison, not just altruism |
| Yin et al., 2017 | Not directly altruism (deception focus) |
| Hu et al., 2017 | Risk/need interaction, not direct altruism |
| Bortolini et al., 2017 | Not direct altruism, focus on group differences |
| Patil et al., 2018 | Anatomy, not state-dependent task or process |
| Niemi et al., 2018 | Types of fairness, not strictly altruism |
| Engen et al., 2018 | Not about altruism evaluation |
| Cui et al., 2018 | Deception study, not altruism |
| Ciaramidaro et al., 2018 | Not altruism—focus on inter-brain connection |
| Brethel-Haurwitz et al., 2018 | Observation only, not altruistic action |
| Kawamichi et al., 2019 | Not directly about altruism |
| X. Xu et al., 2019 | Self-serving focus |
| Lengersdorff et al., 2020 | Focus on pain avoidance, not altruistic motivation |
| Duell et al., 2021 | Focus on prosocial behavior before and after observation, not on altruism |
| N. Zhang & Sun, 2022 | No mention of characteristics of altruistic acts |
| Q. Xu et al., 2023 | No mention of characteristics of altruistic acts |
| Hu et al., 2023 | Mainly about time, context-dependent response speed |
| Y. Zhang et al., 2023 | Target comparison, not altruistic act focus |
| Bak et al., 2024 | Not directly altruism—image valence effect |
| Shi et al., 2024 | Not strictly altruism; emergency vs not focus |
| Ishihara et al., 2024 | Focus on physical activity |
| Gao et al., 2024 | Focus on recipient (not actor), indebtedness |
| Mao et al., 2024 | Empathy focus, not only altruism |

Table S1. Notable studies excluded at the eligibility stage

Bak, S., Yeu, M., Min, D., Lee, J., & Jeong, J. (2024). Charitable crowdfunding donation-intention estimation depending on emotional project images using fNIRS-based functional connectivity. *PLOS ONE*, *19*(5), e0303144. https://doi.org/10.1371/journal.pone.0303144

Bortolini, T., Bado, P., Hoefle, S., Engel, A., Zahn, R., De Oliveira Souza, R., Dreher, J.-C., & Moll, J. (2017). Neural bases of ingroup altruistic motivation in soccer fans. *Scientific Reports*, *7*(1), 16122. https://doi.org/10.1038/s41598-017-15385-7

Brethel-Haurwitz, K. M., Cardinale, E. M., Vekaria, K. M., Robertson, E. L., Walitt, B., VanMeter, J. W., & Marsh, A. A. (2018). Extraordinary Altruists Exhibit Enhanced Self–Other Overlap in Neural Responses to Distress. *Psychological Science*, *29*(10), 1631–1641. https://doi.org/10.1177/0956797618779590

Brunnlieb, C., Nave, G., Camerer, C. F., Schosser, S., Vogt, B., Münte, T. F., & Heldmann, M. (2016). Vasopressin increases human risky cooperative behavior. *Proceedings of the National Academy of Sciences*, *113*(8), 2051–2056. https://doi.org/10.1073/pnas.1518825113

Ciaramidaro, A., Toppi, J., Casper, C., Freitag, C. M., Siniatchkin, M., & Astolfi, L. (2018). Multiple-Brain Connectivity During Third Party Punishment: An EEG Hyperscanning Study. *Scientific Reports*, *8*(1), 6822. https://doi.org/10.1038/s41598-018-24416-w

Cui, F., Wu, S., Wu, H., Wang, C., Jiao, C., & Luo, Y. (2018). Altruistic and self-serving goals modulate behavioral and neural responses in deception. *Social Cognitive and Affective Neuroscience*, *13*(1), 63–71. https://doi.org/10.1093/scan/nsx138

David, B., Hu, Y., Krüger, F., & Weber, B. (2017). Other-regarding attention focus modulates third-party altruistic choice: An fMRI study. *Scientific Reports*, *7*(1), 43024. https://doi.org/10.1038/srep43024

Duell, N., Van Hoorn, J., McCormick, E. M., Prinstein, M. J., & Telzer, E. H. (2021). Hormonal and neural correlates of prosocial conformity in adolescents. *Developmental Cognitive Neuroscience*, *48*, 100936. https://doi.org/10.1016/j.dcn.2021.100936

Engen, H. G., Bernhardt, B. C., Skottnik, L., Ricard, M., & Singer, T. (2018). Structural changes in socio-affective networks: Multi-modal MRI findings in long-term meditation practitioners. *Neuropsychologia*, *116*, 26–33. https://doi.org/10.1016/j.neuropsychologia.2017.08.024

FeldmanHall, O., Dalgleish, T., Evans, D., & Mobbs, D. (2015). Empathic concern drives costly altruism. *NeuroImage*, *105*, 347–356. https://doi.org/10.1016/j.neuroimage.2014.10.043

Gao, X., Jolly, E., Yu, H., Liu, H., Zhou, X., & Chang, L. J. (2024). The psychological, computational, and neural foundations of indebtedness. *Nature Communications*, *15*(1), 68. https://doi.org/10.1038/s41467-023-44286-9

Gospic, K., Sundberg, M., Maeder, J., Fransson, P., Petrovic, P., Isacsson, G., Karlström, A., & Ingvar, M. (2014). Altruism costs—The cheap signal from amygdala. *Social Cognitive and Affective Neuroscience*, *9*(9), 1325–1332. https://doi.org/10.1093/scan/nst118

Hu, J., Konovalov, A., & Ruff, C. C. (2023). A unified neural account of contextual and individual differences in altruism. *eLife*, *12*, e80667. https://doi.org/10.7554/eLife.80667

Hu, J., Li, Y., Yin, Y., Blue, P. R., Yu, H., & Zhou, X. (2017). How do self-interest and other-need interact in the brain to determine altruistic behavior? *NeuroImage*, *157*, 598–611. https://doi.org/10.1016/j.neuroimage.2017.06.040

Ishihara, T., Hashimoto, S., Tamba, N., Hyodo, K., Matsuda, T., & Takagishi, H. (2024). The links between physical activity and prosocial behavior: An fNIRS hyperscanning study. *Cerebral Cortex*, *34*(2), bhad509. https://doi.org/10.1093/cercor/bhad509

Kawamichi, H., Sugawara, S. K., Hamano, Y. H., Makita, K., Kochiyama, T., Kikuchi, Y., Ogino, Y., Saito, S., & Sadato, N. (2019). Prosocial behavior toward estranged persons modulates the interaction between midline cortical structures and the reward system. *Social Neuroscience*, *14*(5), 618–630. https://doi.org/10.1080/17470919.2018.1553797

Lengersdorff, L. L., Wagner, I. C., Lockwood, P. L., & Lamm, C. (2020). When Implicit Prosociality Trumps Selfishness: The Neural Valuation System Underpins More Optimal Choices When Learning to Avoid Harm to Others Than to Oneself. *The Journal of Neuroscience*, *40*(38), 7286–7299. https://doi.org/10.1523/JNEUROSCI.0842-20.2020

Mao, W., Shen, X., Bai, X., & Wang, A. (2024). Neural correlates of empathy in donation decisions: Insights from EEG and machine learning. *Neuroscience*, *564*, 214–225. https://doi.org/10.1016/j.neuroscience.2024.11.044

Mathur, V. A., Harada, T., Lipke, T., & Chiao, J. Y. (2010). Neural basis of extraordinary empathy and altruistic motivation. *NeuroImage*, *51*(4), 1468–1475. https://doi.org/10.1016/j.neuroimage.2010.03.025

Niemi, L., Wasserman, E., & Young, L. (2018). The behavioral and neural signatures of distinct conceptions of fairness. *Social Neuroscience*, *13*(4), 399–415. https://doi.org/10.1080/17470919.2017.1333452

Patil, I., Zanon, M., Novembre, G., Zangrando, N., Chittaro, L., & Silani, G. (2018). Neuroanatomical basis of concern-based altruism in virtual environment. *Neuropsychologia*, *116*, 34–43. https://doi.org/10.1016/j.neuropsychologia.2017.02.015

Rilling, J. K., Goldsmith, D. R., Glenn, A. L., Jairam, M. R., Elfenbein, H. A., Dagenais, J. E., Murdock, C. D., & Pagnoni, G. (2008). The neural correlates of the affective response to unreciprocated cooperation. *Neuropsychologia*, *46*(5), 1256–1266. https://doi.org/10.1016/j.neuropsychologia.2007.11.033

Shi, R., Liu, C., Tang, H., Hao, J., & Shen, W. (2024). Spontaneous giving: Processing mode and emergency affect prosocial behavior. *Acta Psychologica Sinica*, *56*(9), 1239. https://doi.org/10.3724/SP.J.1041.2024.01239

Telzer, E. H., Ichien, N., & Qu, Y. (2015). The ties that bind: Group membership shapes the neural correlates of in-group favoritism. *NeuroImage*, *115*, 42–51. https://doi.org/10.1016/j.neuroimage.2015.04.035

Xu, Q., Hu, J., Qin, Y., Li, G., Zhang, X., & Li, P. (2023). Intention affects fairness processing: Evidence from behavior and representational similarity analysis of event‐related potential signals. *Human Brain Mapping*, *44*(6), 2451–2464. https://doi.org/10.1002/hbm.26223

Xu, X., Liu, C., Zhou, X., Chen, Y., Gao, Z., Zhou, F., Kou, J., Becker, B., & Kendrick, K. M. (2019). Oxytocin Facilitates Self-Serving Rather Than Altruistic Tendencies in Competitive Social Interactions Via Orbitofrontal Cortex. *International Journal of Neuropsychopharmacology*, *22*(8), 501–512. https://doi.org/10.1093/ijnp/pyz028

Yin, L., Hu, Y., Dynowski, D., Li, J., & Weber, B. (2017). The good lies: Altruistic goals modulate processing of deception in the anterior insula. *Human Brain Mapping*, *38*(7), 3675–3690. https://doi.org/10.1002/hbm.23623

Zhang, N., & Sun, X. (2022). Performance Differences Between High and Low Empathy Ability in Conflicts of Interest: An ERP Study. *Psychology Research and Behavior Management*, *Volume 15*, 2979–2987. https://doi.org/10.2147/PRBM.S380838

Zhang, Y., Rong, Y., & Wei, P. (2023). Mothers exhibit higher neural activity in gaining rewards for their children than for themselves. *Social Cognitive and Affective Neuroscience*, *18*(1), nsad048. https://doi.org/10.1093/scan/nsad048
